# Spirit of the Foreign Periodicals, &c.

**Published:** 1842-10-01

**Authors:** 


### Spirit of the Foreign Periodicals.

### Death of M. Double: History of his Last Illness: Funeral Orations.

Among the losses, which the School of French Medicine has of recent years had cause to deplore, there is perhaps none which will be more generally regretted than that of M. *Double*. He was not, indeed, one of those flashy meteors of the day, which dazzle and entrance, but rather a calm steadily-shining star, which, while it adorns the firmament, does not eclipse its fellows by its brightness. And after all, these are by far the best sort of luminaries. They may not attract the gazing eyes of all the multitude, or be the theme of extravagant but transitory wonderment; but, perhaps, for this very reason, they are the more dear to the quiet student, and it is to them that the mariner not unfrequently looks to guide his path along the mighty deep.

M. *Double*, in a silent and unobtrusive manner, raised himself to the very highest honours which the profession could bestow. He was not an original discoverer; but he knew well, and he knew how to appreciate, all the discoveries of others. He was not a prolific author; but he had carefully studied all the best writers, not only of the present day, but also of bye-gone times. He had drunk deeply at the fountain-head; he was an admirable classical scholar, and had imbued his mind largely with the philosophy of such men as *Hippocrates* and *Sydenham*. Our readers cannot have forgotten the beautiful letter which he addressed, about the commencement of the present year, to M. *Dubois*, on the study of the old authors.*

It shewed the character of the man; from the moment of perusing it we seemed to know him better; and many, we dare say, after reading the extracts which we gave of it, formed the resolution of trying to imitate his good example. We ventured at the time to recommend the more general study of ancient medicine, and every day we ourselves feel more and more the advantage of this pursuit. It may not present to the mind an assemblage of so many facts as the writings of modern times profess to do; but it accustoms it to more philosophic thought, to observe better, to reason better, and therefore to practise better. Remember what a first-rate man—was it not John Hunter?—once said of medical science; " there are more false *facts* than false *theories* in it "—a saying pregnant with the most instructive truth, and the perfect accuracy of which is confirmed by every year's experience. For what, pray, is the staple of many works of the present day?—often little more than a mere refutation and denial of the statements made by another author the year before. And then again with regard to the *false facts*, may we not say with perfect truth—without accusing any one with wilful misrepresentation—that there are not a few writers in this country, as well as on the Continent, whose personal testimony would be quite insufficient, in itself, to satisfy us of the accuracy of their observations.

It is only by training the mind to habits of calm and comprehensive reasoning, so that it may avoid, on the one hand, hasty and unwarranted conclusions, on the other, a stubborn incredulity, and that it may know how to discriminate right from wrong, truth from error, to sift evidence, to weigh statements, to test discoveries—and all this without resorting to unjustifiable experiments, whether

* Vide Medico-Chirurgical Review for April, 1842.

in the way of trying new medicines and methods of treatment on men, or of subjecting any of the lower animals to surgical operations—it is only by such a course of study, we say, that the character of a philosophic physician can be attained to.

Now M. *Double* seems to have been such a man; and, when to high professional distinction is added the still higher praise of integrity and right-mindedness in all the relations of life, we have a model before us that all should strive to imitate. We lately gave short biographical sketches of the lives of several other distinguished members of the French school, who have recently died—viz. *Dupuytren, Broussais* and *Richerand,* and we then pointed out what seemed to be the leading defects in the characters of each. All of these men possessed distinguished talents, and succeeded in attracting much public attention during their lives; but not one of them was so fortunate as to win—

> " That which should accompany old age,
> As honour, love, obedience, troops of friends,"

—and what was the reason?—simply this—they did not possess that tranquillity of character that can spring only from inward rectitude; they were all of them slaves of some one passion or another, of envy, jealousy, pride or malice.

But we are wandering away from our proper subject, and will now endeavour, by selecting a few detached extracts from the contents of the French periodicals, to give our readers some idea of M. *Double*'s character.

The following sketch of his last illness was read by M. *Amussat,* at the Royal Academy of Medicine.

" On Tuesday, the 7th of June, M. *Double,* although not feeling quite well, and much distressed by the alarming illness of his brother-in-law, attended the *seance* of the Academy as usual, and afterwards made several professional visits. In the afternoon, while he was sitting in the Duke of Dalmatia's garden, between the marshal and his lady the *marechale,* he felt so uncomfortable a sensation come over him, that at first he thought of retiring; he strove for some time to master it; but in a few minutes afterwards he fell down upon the ground in a state of complete insensibility.

He, however, quickly recovered, and was then conveyed home; but he positively refused to see any medical friend, and even forbad that his children, who were in the country at the time, should be informed of his illness.

I saw him next day; he was in bed, but quite tranquil and composed; he mentioned every thing that had happened on the preceding day, and expressed his opinion that he would be well again soon, especially if he could induce a *crisis* either by the kidneys or by the skin. I urged him to see the physician, under whose care, two years before, he had recovered from a severe pneumonia, and pressed upon him the imprudence of prescribing for himself; but all was in vain. I mentioned to him two cases, which I had seen the day before, of pulmonary congestion, where prompt and decided relief was obtained by bleeding; but nothing would make him yield to my wishes.

Next day, when I called, I was told that he did not wish to see any one: his son informed me that he had perspired freely, that the urine was copious, and that he had expectorated several sputa tinged with blood; this symptom, however, he did not seem to think of any consequence.

On the Friday he still declined any visits, and his state, from his son's account, seems to have been nearly the same as before. On the forenoon of the following day, however, I was admitted to see him; he was lying on a sofa in his study, surrounded by his children. He was in an excited state; he spoke in a short hurried manner, and his features were a good deal altered; the pulse was rapid. I was shewn a clot of nearly pure blood, which he had just expectorated. I wished much to bleed him, but he still refused, and said that he should have it done next day, if he was not better. M. *Roux* came in at this time, and he agreed with me in opinion that the blood came from the parenchyma of the lungs, and that nothing would give so much relief to all his symptoms as venesection.

I saw him again in the afternoon, and found him in nearly the same state; his pulse was still rapid, his tongue harsh and dry, and his articulation somewhat confused. I again urged him to see M. *Andral*; but he still refused.

Next morning he sent for me by seven o'clock to bleed him; he said that he felt a great oppression in his chest, but that he could not say in what point the uneasiness was seated; the pulse at this time was full, large, and beat about 94 in the minute. My wish was that M. *Andral* and myself should have a consultation before any thing was done; but he would not listen to this, and accordingly I bled him without further delay. M. *Andral* called, at my request, in the forenoon, as if *par hazard,* and said to him that he had come, not as a physician, but as a friend to inquire for him: M. *Double* answered at once, 'Je reçois l'ami et le medicin.' He approved of the bleeding—the blood exhibited a decided buffy coat—and advised an emollient enema to be given.

Shortly after M. *Andral*'s taking leave, our patient got up, and walked without any assistance to the dining-parlour, where he took a small cup of semolina boiled in milk, and then returned to his bed-room. His children joined him in the course of a few minutes.

They at once observed a marked change in his appearance; his features were much altered, and his ideas were evidently confused. He complained much of thirst, and the calls to pass urine were very frequent—every time that they returned, he got up and walked to an adjoining cabinet in the passage. When I saw him between one and two o'clock, his eyes were fixed, the mouth was deformed, and his speech was difficult and indistinct; he was now so weak that his son and I were obliged to assist him into bed, after passing water in the standing position.

The symptoms became rapidly worse; the breathing being more and more embarrassed, and the expectoration scanty. At seven M. *Andral* was again called, and ordered a blister to be applied on each thigh, a sinapism on the chest, and an antispasmodic draught to be taken frequently; but every thing proved ineffectual, and he expired about eleven o'clock."

As a matter of course, funeral orations were pronounced over the grave of the deceased. M. *Roux*, the well known chief-surgeon of the Hôtel Dieu, was the first to address the audience.

" Since the loss," said he, " which the Academy sustained in the person of the young naturalist, whose early labours promised so rich a harvest, inexorable death had for a considerable time suspended his rigours amongst us. But, alas! what grief and astonishment has this new stroke not produced! This time, the victim has been one of the representatives of that science which examines and aims to apply the various remedies by which man hopes to ward off the ills which Nature inflicts upon him, and whose great object is to prolong the life of himself and fellow creatures. The colleague, whose mortal remains we have just consigned to the tomb, has been snatched away in the most sudden and unexpected manner, while apparently in perfect health, and when, in full possession of all his moral faculties, and all the activity of his mind, we might have hoped that he would be spared for many years to enrich science with new works, and communicate to the profession which he adorned, the varied results of his immense experience. The elevated character of his mind, the loyalty and genuine nobility of his character, the exquisite perception that he had of every thing lofty, the amenity of his manners, and his invariably dignified behaviour in the exercise of that priesthood to which he had devoted his life, his constancy in friendship, and his ready and generous devotedness on all occasions—combined to constitute one of those moral physiognomies which are as raie as they are truly admirable."

.......... "M. *Double* was born at Verdun (his father was a physician) in 1776. He studied at Montpelier and there took out his doctorate. It was not till 1803 that he fixed himself in Paris, where he was subsequently to rise to such an elevated rank in his profession. He soon distinguished himself by his numerous contributions to a Journal, which was at first called *Recueil Periodique de la Societé de Medecine,* and afterwards *Journal Generale de Medecine,*—for the first twenty-five years of the present century the leading medical periodical in Paris—edited by M. *Sedillot,* one of the young physician's earliest and best friends. M. *Double* subsequently became editor himself, and for seven or eight years superintended the Journal with great assiduity and success. He must have devoted a very large portion of his time to this task, as most of the reviews of new works, the regular reports of the medical constitution of the metropolis, and many original memoirs on subjects of practical medinice were written by himself.

No doubt it was while engaged in the labours of his editorial duties, that he acquired that vast literary knowledge of his profession, which he afterwards turned to such admirable account in following out its practical duties. He was an excellent scholar; cared little for the ordinary pleasures of the world, and devoted himself almost exclusively to the enjoyment of his library, and the quiet recreation of domestic and friendly life. Be it remembered that M. *Double* was never attached to any hospital or large public institution; perhaps the attainment of such a situation was not much coveted by him, as his time must have been fully occupied with the labours of his study, and the demands of his gradually increasing private practice. He was one of those men," adds M. *Roux,* "unfortunately too rarely met with, who, although ambitious of rising high in his profession, knew how to set bounds to his desires. He became member, and subsequently president of the Academy of Medicine, member also of the Academy of Sciences; the duties of these honorary situations as well as the constant demands of his private 'clientelle,' left him not more than time to devote to his favorite pursuits of literature, and the still more engaging demands of parental instruction. Not many years after his marriage he had the misfortune to lose his beloved companion; subsequently his two children became the chief objects of his unceasing regards; and he had his reward; for seldom have a father's hopes and pride been more amply gratified."....
...................... "M. *Double* competed for the famous prize offered by *Napoleon* for the best Essay on Croup; and, although his was not the successful one, it was so fortunate as to receive 'the first honourable mention.' It was published, and is still regarded as a very valuable monograph on the subject. Not long afterwards he brought out his elaborate work on *Semeiology* or the Doctrine of Symptoms as indicative of the nature and probable termination of diseases. It is a work of great compass of thought and observation, and is replete with valuable instruction to the practical physician.

Fortunately for M. *Double* he was associated with his father-in-law, M. *Pelletier*—father of the present distinguished chemist of that name—in the examination of the chemical and physiological properties of the vegetable alkaloids. M. *Pelletier* had succeeded in preparing a considerable quantity of the substance, now so universally known by the name of quinine, and he engaged M. *Double* to ascertain its remedial virtues, and to determine how far it may be depended upon as a substitute for bark. It is unnecessary to say more upon this subject than merely to state that M. *Double's* memoirs to the Academy established the efficacy of the alkaloid beyond all doubt, and that the MM. *Pelletier* have continued ever since to retain their well-deserved reputation for the admirable quality of their preparation."...............

"I could mention," says M. *Roux,* "many examples to prove the more than ordinary high-mindedness, and strict integrity of character of the man whose friendship I enjoyed for nearly forty years, and whose counsel on almost every occasion I prized so highly. While still a youth, he accompanied his brother—

who, from being the bishop of Tarbes, was obliged to exile himself from France, during the storm of the first revolution, into Spain,—and continued to remain with him in the prison of Figueras, that he might share and relieve his distresses.

We all know the noble part he acted when a peerage was offered to his acceptance:—proud, and justly proud, was he of this intended honour; but 'never,' said he, 'can I accept it on the terms of renouncing that profession to which I have been indebted for every thing I possess.'

Not long before his lamented decease, a body of the electors of his *arrondissement* waited upon him to request his permission to be proposed as the Deputy at the ensuing election. 'I would gladly accept the honour, if the suffrages of the majority be spontaneously offered to me, and proud should I be of the great mark of my fellow-citizens' esteem; but I shall never go about soliciting their votes. I know that my saying this is equivalent to my declining the offer; for my mode of acting does not suit the manners of the present time or of this nation.' "

The close of M. *Roux's* address was simple and touching—at least, considering the usual style of funeral orations in France.

" Such a man, so highly gifted with moral and intellectual endowments, will long be remembered and regretted by all who knew him. Some may be inclined to think, that I have not said enough in his praise, and that I have spoken of his good qualities with too much coldness and reserve: it may be so; for, while addressing you, I could not get rid of the idea that he was standing beside me; and well I knew how he disliked to hear any adulation or praise offered to him; he would always reproach those who did so. Would, oh! beloved friend, that my voice could now reach you!—how sweet were our mutual interchanges of thought and feeling!—and what a sad blank has your death for ever caused in my existence! But it cannot be for long that I will have to deplore your loss: a few months—what do I say? perhaps only a few days, or at most a year or two, and we shall again meet in the eternal mansions. Adieu."

M. *Guerin* followed M. *Roux*, and "saluait l'ombre" of the deceased in rather more ambitious oratory. He spoke of the "grande douleur" in the temples of science, and of the "immense sensation" which the news of M. *Double's* death occasioned among all the medical men in Paris, "whom I," said the speaker, "though sprung but yesterday from the plebeian rank—(is this of science or of parentage?—*Rev.*)—am called upon to represent, and in whose name I now lay upon the tomb their homages and grief and their last adieu." M. *Guerin* in his discourse alluded particularly to the extreme quietude, so to speak, of M. *Double's* professional career; his name was never brought very conspicuously before the public either as a writer or speaker, and, as we have already mentioned, he was never a physician of any hospital, nor was he distinguished for any brilliant discovery or important scientific work; and yet here is an example, said M. *Guerin*, of a man raising himself not only to the highest rank in his profession, but admitted a member of the Academy of Sciences, which is well known to be jealous of its diploma except to those who have made themselves famous by their inventions or discoveries. It may be worth while to mention that the rival candidate on this occasion, over whom he succeeded by a few votes, was "le medecin le plus celebre de l'epoque, l'homme qui avait revolutionné la science et repandu son nom d'un bout de l'Europe à l'autré"—can this be aught than poor *Broussais* whose "decadence" has been nearly as great as his rise. It is certainly creditable to M. *Guerin's* candour, who, we need scarcely say, is a mighty admirer of the Napoleon of Medicine, as some of his ultra-admirers have called him, to find that he admits the perfect fairness of the Academy of Sciences when they gave the preference to M. *Double.* He says: "It was not to any accidental circumstances, to any caprice of chance, or to any merely superior tact, that we can trace the almost constant success which attended his career, and which raised him from the most modest ranks to the very summit of professional and scientific distinction. Now what was the cause of this? It is perhaps not very easy to designate it in a few words; but there is no reason that we should attribute it to mere good luck or happy fortune. There was an unbroken continuity of success in the career of M. *Double* which must have struck every observer; he triumphed not upon one, but upon all occasions; not only in the Academy of Medicine, but also in the Academy of Sciences; in his profession, and in the daily affairs of life. Was it not to him that the Academy almost always looked in all delicate and difficult circumstances? and who does not remember the courteous dignity with which he uniformly discharged the duties of the president's chair? How often when the over-vivacity or confusion of the members threatened to disturb the deliberations of this 'docte assemblée,' would he with a single word conciliate every difference, restore light where hitherto all was dark, and bring to a close what seemed to be interminable! When the pestilential cholera threatened our shores, it was he that was appointed to determine the value of our resources to oppose its progress; when the judicial courts appealed to the Academy for its opinion in a memorable case involving the responsibility of medical men, it was he who was named to draw up the terms of reply; when Government called upon it to present a plan for the re-organization of the profession, it was M. *Double* again who was entrusted with this important task; and when there was an intention of elevating a physician to the peerage, it was he whom the whole medical corps designated as the most worthy to receive that honour. In his private practice it is well known that he was the intimate confidant of many of the most distinguished men of the day; and no one had ever to regret the unbosoming of his mind to him."

M. *Guerin* is quite right in saying that such eminent distinction as M. *Double* attained to, or that the universal esteem in which he was held by all his equals and rivals, could not possibly be the result of any surprise, or any mere temporary cause. It was the fruit of a long life of high integrity and unwavering consistency. "To a truly elevated spirit, a clear judgment, and a profound experience of men and the world, he added a nobleness of heart, great firmness of character, a well-poised moderation in all his actions, and a deep and sincere love of humanity. He possessed in a remarkable degree a 'veritable genie de conduite;' and we need scarcely say, his life was uniformly pure and honorable."

---

### M. DUMAS ON THE CHEMISTRY OF ORGANISED BEINGS.

It appears that MM. *Dumas* and *Boussingault*, in France, have been following out nearly the same pursuits in Organic Chemistry, as Professor *Liebig* has been doing in Germany. It is immaterial to us to whom the priority belongs; the important point for science is, that these gentlemen have arrived at very similar conclusions.

From a recently published memoir of M. *Dumas*, we shall select a few passages that bear more immediately on the physiology of plants and animals.

"We have," says he, "considered *plants* as constituting an immense reducing or decomposing apparatus, that is nourished by carbon, hydrogen, and nitrogen—derived from the decomposition of carbonic acid, water, and oxide of ammonium—and *animals* as forming a large consuming apparatus, in which there is constantly going on the combustion of these elements, carbon, hydrogen, and ammonium, to form these very compounds.

We have laid it down as a recognised principle that plants form, or prepare, from mineral substances the organic materials of their composition, that these materials are taken into the bodies of animals, are there subjected to the process of digestion, thus become animalised, and are subsequently again brought back by a vital process to the state of mineral and inorganised matter.

As accessory results of our researches, we may state that some plants absorb a certain portion of nitrogen from the atmophere, while others do not; that animal heat is owing solely to respiration; that the chemical process of this function (respiration) takes place not in the lungs but in the capillary vessels of the whole body; that digestion effects two important results, viz. the assimilation of azotised matters, and the restitution of combustible matters to the blood."

M. *Dumas* then discusses at considerable length the striking effects of ammonia in promoting vegetation: this seems to be the potent agent in most manures. Hence whatever tends to assist the formation of this substance, or to render it more fixed and abiding, is found to increase the valuable properties of manures. The addition of a solution of sulphate of iron, or of weak sulphuric acid, has this effect, and has been found to add greatly to the fertilising properties of these matters. A sulphate of ammonia is formed, and remains fixed; the salt being not volatile like its alkaline basis.

With respect to the much vexed question, as to the source of animal heat, it seems to be the opinion of M. *Dumas,* that during each act of respiration a certain portion of oxygen is absorbed directly into the blood, and a certain portion of carbonic acid—already formed and existing in the blood—displaced and evolved. There is no direct union or combustion, so to speak, of hydrogen or carbon with oxygen in the lungs, as imagined by *Lavoisier* and *Laplace*: the formation of the carbonic acid seems to be a slow and successive act that is constantly going on in the minute blood-vessels; and the venous blood, when it reaches the right side of the heart, is already charged with it, and ready to give it off when exposed to the air in the cells of the lungs.

It is necessary to keep in mind that the production of animal heat, the exhalation of carbonic acid into, and the disappearance of oxygen from, the respired air are three separate phenomena—connected, indeed, the one with the other, but not implying that they are of simultaneous occurrence. Or we may express our meaning in different words thus: the generation of animal heat, the decarbonization, and the subsequent arterialization, of the blood, are three mutually-associated, but not coincident, phenomena. The blood becomes arterialised without any necessary production of heat at the time: and the gradual formation of carbonic acid—an act which is necessarily attended with the evolution of caloric—is going on in every capillary vessel throughout the body.

" Respiration," says our author, " introduces oxygen into the blood and renders it of a bright red colour; carbonic acid is at the same time expelled from it.

*L'oxygene absorbé sert à bruler du lactate de soude, et en general des sels de soude. L'acide lactique transforme celui-ci en lactate et degage acide carbonique. Cet acide lactique provient des alimens sucrés ou amylacés.*"

" The fatty matters also form salts with the soda of the blood. When they are in excess, they become deposited around the vessels through which they have exsuded, and, by being blended with the albuminous fluids in a state of repose, they serve for the production of adipose cellules or vesicles, and the animal becomes fat—in other words, it lays up a supply of combustible matter. When the organised materials of the blood are consumed (brulées) without being replaced, the vital fluid becomes more decidedly alkaline and begins to react upon the adipose vesicles which surround the vessels; the fat and albumen of the cellules is re-dissolved and absorbed by the blood, and the animal becomes emaciated—in other words, it consumes the combustible matter which had been stored up at a former time."

M. *Dumas* closes his remarks on this subject by pointing out the difference in the various kinds of food, according to their mode of action on the system and the changes they undergo during digestion and assimilation. He arranges them in three classes :—

1. Aliments of assimilation—viz. fibrine, albumen, and caseum : these are all of a highly azotised nature.

2. Soluble non-azotised aliments of respiration : such as starch, sugar, acid or acidifiable substances, &c., which at once undergo combustion in presence of the soda in the blood ;—hence the production of heat manifested from the very commencement of their digestion.

3. Aliments of respiration, insoluble and therefore capable of being stored up in the body, viz. various kinds of fatty matter."—*Annales de Chimie.*

We may here insert a paragraph or two from M. *Liebig's* lecture on the different kinds of food.

" Another most interesting result of M. *Liebig's* researches is that vegetable albumen, fibrine, and caseine not only have the same properties as the corresponding elements derived from animal matters, but also exhibit their azote and carbon in the same relations to each other. Thus chemical analysis shews us that herbivorous animals find the constituent materials of their blood, their albumen and their fibrine, already prepared in plants; and that the juice of plants, the vegetable albumen, the farina of wheat and of other *cerealia* contain the principle of muscular fibre, while lentiles, peas and beans, contain the same azotised substance that is present in milk. They (herbivorous animals) live upon the flesh, blood, and cheese supplied them by plants; while, on the other hand, their flesh and blood serve for food to the carnivorous tribe. There is thus a complete identity between the azotised principles existing in vegetables and those in animal substances. Their chemical properties are alike; for we find vegetable albumen, obtained by boiling the juice of plants, and freed from all fatty and colouring matter by means of ether and alcohol, can scarcely be distinguished from the white of eggs."—*Algemeine Zeitung.*

---

### M. LONGET AND HIS VIVISECTIONS AGAIN!

We observe that the French Academy has again been entertained with an account of some new experiments performed by this dog-killing empiric to ascertain "the influence of the pneumo-gastric nerves on the movements of the stomach." Have we not known for the last two, or perhaps for the last twenty, centuries, that the nervi vagi preside over the function of digestion, and that wounds or diseases affecting them inevitably bring on some derangement or another of the stomach?; and are not the experiments of *Wilson Philip*, even if we had no others, amply sufficient to satisfy the most sceptical? It would seem not—judging from the recent goings on of M. *Longet* and some others of the experimental gentry of the French school. Indeed we much fear that the poor canine race will continue to be sacrificed year after year by these shallow sciolists, who seem to be utterly ignorant of what has been done before them, and foolishly imagine that it was reserved for them to lay the foundation of physiology. The most lamentable feature of all is, that not a single new truth is ever discovered, or a single acquisition of any importance made to genuine science, after this lavish and most unjustifiable destruction of animal life.

Year after year, nay generation after generation passes away, and, in spite of all the advantages of modern times, the discovery of any new truth in physiological science is, it must be confessed, a phenomenon of exceedingly rare occurrence. This is certainly from no want of zeal or enterprise on the part of its votaries; for never has there been so much officious assiduity among any set of men as among the physiologists—more especially those of the French school—of the present century. Great has been the labour, but little is the harvest to shew for it all—or, to use the northern proverb, we may say, it has been "a' cry, nae woo." We verily believe that such men as *Bichat* in France and *Hunter* in England knew much more of the laws of animal life, and communicated to their pupils much sounder and more scientific principles than any professor of the present age, notwithstanding the valuable aids which we enjoy from the important discoveries—*real* discoveries these truly are—which have been made since their time in many of the necessary sciences—such as chemistry, botany, comparative anatomy, &c. The cause of this is that we have been on a wrong track almost ever since their days; experiments on living animals have taken the place of studious observations on the phenomena of vitality exhibited by Nature in disease and in health, and of the careful comparison of the forms of organization and the functions displayed in different classes of animals. Until we return to this plan of enquiry, we can expect little more than an ever-renewed succession of fruitless and most cruel operations, and of visionary and absurd speculations. Have we not been told, within the last 20 or 30 years, that the greater portion of the brain may be removed from some animals without much seeming injury to them—that if one portion be irritated the animal runs forward; if another, it runs backward; and if a third, that it pirouettes about or makes somersets in the air—that the optic nerves are not the nerves of vision, and the auditory not those of hearing—that an animal will vomit nearly as well with a wet bladder, secured to its œsophagus and duodenum, as with its natural stomach—that the system of the absorbent vessels is almost an unnecessary and supererogatory appendage to the body, as the veins can perform all their duties quite as well—that the epiglottis is of very little utility, as the act of swallowing and the use of the voice are scarcely, if at all disturbed, if this appendage be cut away? A host of other cases might be alluded to, where the most extravagant absurdities have been gravely proposed and most elaborately discussed both by word and by pen among physicians of all countries. Take, for example, even that much vaunted topic of cardiac physiology—the movements and sounds of the heart. To speak the simple truth, we must confess that we scarcely know a whit more about the subject than when *Laennec* first drew our attention more specially to to it. One theory has given way to another, this to be replaced by a third, until a fourth or fifth, equally short-lived, made its appearance. Oh! for one short year of such men as *Harvey*, or *Haller*, or *Hunter*, to set us again in the right path of enquiry, and to dissipate those flimsy clouds—"clouds without water, carried about by the winds"—of modern speculation!

But to return *à nos moutons*, we shall give one short extract from the report of M. *Longet's* memoir, read before the Academy.

"Having laid open upwards of 40 dogs, he ascertained that in the greater number of them the irritation of the pneumo-gastric nerves induced evident contractions of the stomach—which, in many instances, seemed to be as it were strangled round its middle. The degree of this strangulation appeared to be dependent upon the state of the stomach at the time, whether digestion was going on or not. In the former case, irritation of the pneumo-gastric nerves induced very striking contractions of the viscus; but these were less conspicuous when the process had nearly or quite ceased.* It has been no doubt from neglecting to attend to these circumstances that writers have differed so much among themselves in the results of their experiments upon these nerves, and the conclusions which they have drawn from them.

---

* Very surprising certainly!—just as if we were gravely told that the application of any irritant to the rectum or bladder induced stronger contractions in these viscera during their expulsive efforts than when they were quiescent—rare discovery of M. *Longet!*

M. *Longet* also ascertained that the effects of irritating the pneumogastric nerves are almost always the more decided and conspicuous, the nearer to the stomach that the irritation is applied.

No contractions or movements of this viscus seemed to be induced by acting, either with mechanical stimuli or with galvanism, on the twigs of the great sympathetic."—*Encyclographie des Sc. Medical.*

We now ask our readers whether we are justified in stigmatising the whole series of M. *Longet's* enquiries,—to use the elegant language applied by the Member for all Ireland to his old friends the Whigs—as "bloody, base, and brutal."

---

### ON THE MOVEMENTS AND SOUNDS OF THE HEART: REPORT TO THE BRITISH ASSOCIATION, &c.

We do not pretend to understand the meaning of several of the following sentences, but we give them as we find them, without "extenuating or setting down aught in malice." The subject of cardiac acoustics—if we may use such a phrase—is certainly not easily comprehended; and little is the wonder; for not a half-year passes over our heads, but some new theory or another of the sounds of the heart is proposed to replace its predecessor—which, as a matter of course, is judged to be entirely wrong.

"I have been led," says a recent correspondent of the Royal Academy of Medicine, "by the comparative examination of the heart in the human subject and in quadrupeds, and by studying the arrangement of the muscular tissue of the organ itself, to conclude that the heart turns upon itself or rather becomes twisted (se tordait) during its systole, and untwisted (se detordait) during its diastole. This circumstance I have been able to ascertain, 1, in animals, by exposing the heart and planting in it long needles, which were then observed to describe a quarter of a circle at each contraction; and 2, in the human subject, especially when the person is very lean, and has hypertrophy of the heart, by placing the forefinger of each hand on the precordial region, where the pulsations of the heart are felt; for then the finger that is nearest to the sternum will be observed always to feel the impulsion first.

The movements of the heart consist, 1, of a twisting movement (mouvement de torsion) from right to left, of the rising of the apex, at first in the same direction, and afterwards directly from below upwards; 2, of an untwisting (mouvement de detorsion) from left to right; and of the falling down of the apex.

The mode in which the ventricular cavities are closed and dilated is much the same as what we observe in twisting and untwisting the finger of a glove, held by its tip, and the aperture of which is kept open by means of a ring: during the *torsion*, this cavity is obstructed; and, on the contrary, during the movement of *detorsion* it is dilated.

The right ventricle, during the act of contraction, rolls over towards the left (se roule en partie sur le gauche): both cavities contract at the same time, unless indeed there is any obstruction at the orifice of the aorta or of the pulmonary artery; and then the corresponding ventricle is longer of discharging its contents than the other.

The double sound or tictac of the heart's action seems to be produced by the alternate adhesion and separation of the opposite sides of the ventricular cavities; the first sound being produced by the former and the second by the latter state.*

---

* To prevent the possibility of our mistaking the author's meaning, we give

The cavities of the auricles, being only imperfectly obliterated during their contraction, give rise to a sound so feeble that it can scarcely, if at all, be heard in in the state of health; but, in certain cases of disease, it becomes perceptible, and then it is observed to precede the first of the ventricular sounds.—*Gazette Medicale.*

We may as well take this opportunity of making some allusion to the last report, which the committee appointed by the British Association has published a twelvemonth or more ago.

The reporter was Dr. *Clendinning,* and he seems to have been the prominent and most active member of the committee. We wish that we could have approved of the general plan and manner in which the enquiry was carried on; but this we cannot do; they savour far too much of the bad spirit of the modern French school,—which may certainly with justice call itself *experimental,* as its votaries seem to do little else but make experiments at the expence of animal life. The series of experiments performed by Dr. *Clendinning* and his associates was carried on at intervals during the years 1839 and 1840: young donkeys were the chief sufferers.

The object of the experiments seems to have been twofold—1, to ascertain the causes of the various abnormal sounds attending the heart's action in disease; and 2, to discover how far the motions and sounds of the heart in the lower animals correspond with those in the human subject.

The first point was *endeavoured* to be solved by attempting to imitate artificially several of the morbid conditions, to which the heart is spontaneously liable, either by the application of stimuli to its surface, so as to excite inflammatory action and its consequences, or by mechanically displacing it, or the large vessels which issue from it, so as to interrupt to a greater or less degree the free circulation of the blood.

For these purposes, a multitude of experiments—many of them cruel, and almost all of them, in our opinion, quite unnecessary—were performed on young asses and other animals. For example, long needles and slender trochars were pushed into the chest in the direction of the heart so as to pierce its substance—"the instrument then exhibited motions corresponding to those of the heart"—and then liquor ammoniæ and other irritating fluids were introduced into the pericardium. The changes in the sounds and movements of the heart were, as a matter of course, most scientifically examined and registered each day, until the poor animal died; and then a regular *inspectio cadaveris* took place, every appearance being duly noted.

We observe, by the bye, that in one of these experiments it is stated that, when the needle was introduced in the direction of the heart for about three inches, "it presented rhythmical movements sternad and dorsad: the latter being slow and forcible, and synchronous with the first sound, and the former being sudden, like a fall-back from gravitation, and accompanying the second sound."

Is not this statement at variance with the generally admitted opinion that the first sound is synchronous with the impulse of the heart, and consequently with its movement forwards or sternad?

In some of the experiments a long curved tenaculum was introduced in the direction of the great vessels, and moved about in different directions, so as sometimes "to draw at the root of the arteries," and then to "relax the hold;"—with the view of ascertaining what changes the cardiac sounds might expe-

---

the words of the original:—"Les parois internes du cœur s'appliquant avec force les unes contre les autres produisent, au moment òu elles se touchent, un bruit qui est le premier temps du tictac normal; lorsqu' elles se separent, un second bruit qui est le deuxieme temps."

rience. All this certainly appears to us to be as rude and coarse a way of investigating the phenomena of life, as can be well imagined. What good can possibly be anticipated from such mechanical manipulations?

Then again what useful inferences can we draw from such an experiment as the following? The animal being pithed, and artificial respiration being kept up, the chest was opened and the heart exposed. "On introducing a finger into the right auriculo-ventricular orifice, the first sound was accompanied with a whiz, and wanted its flapping beginning. The whiz was accompanied by a thrill sensible to the finger. The whiz ceased, and the systolic flap returned on removing the finger."

Again, in some of the experiments, threads were passed through the appendices of the auricles, and being held tense so as to impede the auricular systole, were felt to be drawn downwards with much energy immediately before the systole of the ventricles, auricular contraction being completed, while the ventricles were still developing their systole."

All this, it seems to us, is in the very worst manner of the modern experimental physiologists of the French school.

But our readers—who may not have perused the original communications of Dr. *Clendinning*—may ask what is "the conclusion of the whole matter," after so much learned labour had been expended. In reference to the cardiac sounds, the chief inference seems to be that the first sound is attributable, "in part, to the abrupt closure and transitory tension of the auriculo-ventricular valves, but chiefly to cardiac muscular tension:"—*tension* is surely not the proper word here; the fibres of a muscle, during its contraction cannot well be said to be *tense*, as they are then rather *shortened* than otherwise. It seems rather strange that no distinct or definite attempt at explanation of the second sound is given. We gather, however, the opinion of Dr. *Clendinning* on this point—and it is probably the right one—from the last conclusion, which we give entire, for a two-fold reason.

"That the sounds of the heart, like the motions, are governed by the same law in all warm-blooded animals hitherto examined, and probably in all kinds whatsoever;—viz. that the *first* sound in all animals is longer and obtuser, and the *second* sound shorter and sharper; that these sounds are, as in the human heart, respectively systolic and diastolic; that their causation likewise follows the same law as those of man, the *first* sound being mainly muscular, and the *second* exclusively valvular—(the closure, we suppose, of the semilunar valves); —likewise that there is the same causation and mutual relation of the cardiac and arterial pulsations."

There seems to be scarcely a single point in the physiology of the heart's action that has not been, and alas! still remains, a subject of difference of opinion. For example, medical men are not agreed among themselves whether the movements of the auricles and ventricles strictly alternate with each other, or whether they are continuous and immediately successive; whether the systole of the auricles contributes to the propulsion of the blood; whether the elongation and shortening of the ventricles occur during their systole or their diastole; what is the cause of the pulsation or impulse of the heart against the side; whether there is any power of suction exercised by the cardiac cavities, during their diastole, on the blood contained within the large veins; whether the systole of the auricles is attended with any perceptible sound; and, lastly, what is the cause of the double sound of the healthy heart—not to mention the various abnormal sounds which are induced by the existence of various diseases!

We believe that our brethren of the long robe congratulate themselves occasionally by toasting "the glorious uncertainty of the law." We much fear that theirs is not the only profession to which "uncertainty" belongs—with ours, however, it is any thing but "glorious." And yet it is not so much to the very nature of our pursuits, as it is to the mode of our following them out, that the humiliating unstableness of many branches of medical science is to be traced. We medical men must be taught the elements of sounder logic, and learn to exercise a little more of that rather rare endowment "common sense," before we can hope to make many steps in advance. Certainly it is not by merely laying bare "the bloody house of life," and rudely looking at its moving machinery, that we shall ever fathom its higher mysteries. We verily believe that there is a curse on the wilful sacrifice of animal life, except for the purposes of food to mankind. "Every moving thing that liveth shall be meat for you,"—this is sufficiently explicit; but where is the warrant to kill for the sake of science?—*(Rev.)*

---

### Influence of Tobacco on Phthisis.

"*To the Editor of the Gazette Medicale.*

Sir,

In 1836, I published a paper on the influence of tobacco on the health of the workmen in the Royal Manufactories. I there put forth the following statement: 'Pulmonary consumption is rare among the workmen, who are engaged from their youth in the manipulation of tobacco; moreover, this disease makes much less rapid progress than it does usually in those who may happen to have the germ of it already developed when they enter the workshops.' My experience during the last six years has amply confirmed the accuracy of this statement, and, thanks to the improved organisation of the medical service in the Royal Manufactories, I am now able to collect materials more easily than hitherto. My only motive, at present, is to direct the attention of medical men in general to the circumstance now mentioned, and to solicit them to examine the question for themselves.

Maurice Ruef.

*Strasbourg,* 31st May, 1842.

(The hint is a good one: the soothing effects of tobacco-smoking, where it does agree, may be really very useful in cases of incipient or threatened phthisis.—*(Rev.)*

---

### History of an Epidemic Meningitis; followed by Comments on French Medicine.

M. *Forget,* Professor of Medicine in the School at Strasbourg, has written a series of papers in the French Medical Gazette, on a peculiar epidemic disease which prevailed during the course of last year in that city. These papers are so elaborately minute, that we find considerable difficulty in giving the "pith and vital sap" of them—for this is all that we aim to do—they are so overlaid with wordy details, thick and frequent as the leaves in Vallombrosa.

We pass over, therefore, his prefatory remarks on the importance of medical speculations, the uncertainty of medical nomenclature, the difficulty of many medical inquiries, and so forth.

One of his objects seems to be an endeavour to shew that there is nothing necessarily peculiar or specific in a disease from the mere circumstance of its being epidemic—*i. e.* wide-spread over a greater or less extent of population;—that it still remains essentially the same malady, requiring the same mode of treatment, as when it occurs sporadically and in isolated cases. He alludes to the sad and flagrant errors into which most medical men are apt to fall upon the apparition of any novel epidemic. "Witness," says he, "the lamentable mistakes they committed, when alarmed and bewildered by the invasion of the oriental cholera, and the truly *bizarre* notions that have been entertained on the subject of the *grippe* (the influenza)—and yet what has been the result of our experience of these two epidemics? neither more nor less than that the same treatment, to which we trust in cases of sporadic cholera and bronchitis, is applicable to them."

Now this, like almost every other sweeping and wide-embracing assertion in medicine, is partly true and partly untrue. We *do* say that whenever a disease becomes decidedly and extensively epidemic—be it the influenza or the cholera, or typhus fever, &c.—its *type* (to use an old medical term), or prevailing character, will be found to be really and intrinsically more or less modified. Has it not been observed, as a very general fact, that cases of the genuine epidemic influenza will not stand bleeding and other lowering remedies so well as those of simple and occasional catarrh? and where is the medical man that will be so bold as to assert that the treatment of the pestilential cholera is similar to, but only more energetic than, that of ordinary Summer purging and vomiting. Not many will go so far; the latter is a local, the former is a universal systemic, disease; the latter is the result of an overflow or an acrid state of the bile and other intestinal secretions, the former is the result of a poisoning and vitiating of the blood and all the humours; the one belongs to the class of *profluviæ*, the other is essentially a pestilential and malignant fever. But it is unnecessary to pursue this subject any further; our only object in these passing remarks being to guard any of our readers against too readily admitting what are called "general principles" in medicine. It has been from the neglect of this caution that the French school in particular has, especially during the present century, fallen into a succession of the most fallacious errors in the treatment of diseases.

But to come to the immediate subject of our inquiry—the epidemic meningitis of Strasbourg—we are told by M. *Forget*, that in the latter months of the year 1840, several cases of severe encephalic disorder were observed in the military hospital of that city; but that it was not until the last week or two of the year that their number became so rapidly frequent as to excite marked attention. From this time to the beginning of May in the following year, the epidemic seems to have continued; although during this time several distinct remissions or periods of abatement were observed. Confined at first to a single regiment, it soon attacked the other corps of the garrison, decimating the soldiers, but generally sparing the officers.

It had existed for a considerable period among the troops in the garrison, before the inhabitants of the city became affected; and it was therefore for some time imagined that the diet of the soldiers might have something to do with it: its subsequent progress, however, quite contradicted this idea. The weather, too, seems to have had comparatively very little influence either on its development or its career: it made its first appearance in Winter—whereas ordinary encephalitis is much more frequent in hot than in cold weather—and continued throughout the subsequent Spring to nearly the middle of Summer. M. *Forget* admits that, after the most patient examination of the subject, he could not trace the disease to any of the usual exciting causes of inflammatory action, such as exposure to cold and wet, irregularities of diet, &c. &c. and that he is therefore obliged to infer that it, like influenza, cholera, and other epidemics, must be referred to some specific, although unknown, conditions of the atmosphere. While thus admitting the atmospheric origin of the disease, he does not believe in its contagiousness, or direct communicability from one sick person to another. None of the nurses, or even of the other patients in the hospital, seemed ever to catch the disease from any patient, who was admitted while labouring under it.

(Upon the whole, this seems to be generally the case with most regular epidemics; as for example cholera, influenza, erysipelas, &c. It is rarely, if ever that we meet with a case of such diseases, where we can unhesitatingly pronounce that it has been transmitted from one person to another. That however the bodies of patients labouring under them may emit effluvia which, under certain, —although to us unknown—conditions of the air will favour the development of the same disease in the attendants, seems to us to be quite warranted by the results of experience, and to be in strict harmony with the history of the genuine contagious diseases. As, in most matters connected with medical science, the truth here seems to be in the *juste milieu,* equally retired from the extremes of an over-ready credulity, that willingly adopts any view of the subject which saves the trouble of much enquiry or discussion, and of a cold rigid scepticism, which will admit nothing but what can be made a matter of sensual proof.)

It is not very easy to make out exactly what are really the opinions of M. *Forget* on some points. While he tells us that the epidemic encephalitis, of which he is treating, has this peculiarity, that its inducing causes are certainly not those of the ordinary spasdic form of the disease, he follows up this statement by saying that he nevertheless sees no reason why they should not be regarded exactly in the same light and treated altogether in the same manner. His reasoning will be seen from the following passage :—

"No doubt the existing cause of the disease is strange; but is this any reason for supposing that it produces a *specific* affection? Does pneumonia from the inhalation of an irritating gas differ in any respect from pneumonia induced by other causes? Admitting—and remember that we are completely ignorant of the subject—that the cause is entirely different from the ordinary exciting causes of the disease, does it follow from this that it shall necessarily give rise to unusual and strange results?"

We (Rev.) have already, in some of the preceding remarks, expressed our opinion, which differs certainly very materially from that expressed by M. *Forget.* He believes that the ætiology of a disease, or the mode in which it has been generated and developed, has very little influence on its type or character, and that it need therefore scarcely be taken into account in determining our plan of treatment. Here we are issue with him. We think that the type, and therefore the *ratio medendi* also of a disease are greatly influenced by the cause which has given rise to it—and more especially whether this be epidemic and *seasonal,* or whether it be merely casual and sporadic. As a general remark, we should say that diseases, when decidedly epidemic, very usually induce a greater degree of debility and prostration of the entire system, than when they are sporadic. Is not this the case with genuine influenza, when compared with casual bronchitis? and with epidemic compared with casual erysipelas? Even with respect to those diseases which are decidedly contagious, as well as generally epidemic, the same remark usually holds true. A casual sporadic case of small-pox, or of measles, is generally of a more phlogistic type than the average number of cases during an epidemic prevalence of the disease. But even in the case of sporadic diseases we contend that their type is often very much modified by the course which has produced them. To take the very example which M. *Forget* adduces, viz.: pneumonia, when induced by the inhalation of an irritating gas, and pneumonia supervening upon exposure to wet and cold. It may be necessary to premise that, in our opinion, almost every case of ordinary genuine inflammation, more especially when any important organ is affected, is preceded for several days or even weeks by a state, so to speak, of *incubation,* in which the blood is undergoing certain changes, which lay the foundation for the explosion of the disease. It seems to us to be a great mistake to suppose—although the idea is a very common one—that attacks of pneumonia, pleuritis, rheumatism or peritonitis are often very sudden in their occurrence, and that they supervene in the course of a few hours after a sudden chill of the body. This is a very general supposition among unprofessional folks; and even not a few medical men also allow themselves to be carried away with it. And yet experience certainly does not warrant it. In ten cases out of a dozen, it will be found that the patient has been more or less indisposed—" out of sorts"—for a week or two probably, or it may be longer, before the inflammatory attack came on. There has been a certain degree of feverish state of the system, not sufficient perhaps to make the person keep the house, but which had shewn itself by tolerably obvious symptoms—such as loss or irregularity of appetite, more thirst than usual, lassitude and inaptitude for ordinary bodily and mental exertion, disturbed sleep, probably headache, along with constipated bowels (generally) and deep-coloured scanty urine (always). Now it is during this state—which may be called the period of *incubation*—that the blood is gradually undergoing those preliminary changes which precede the explosion of the inflammatory attack. In what other way can we reasonably account for those characteristic deviations in the condition of the blood from the standard of health which exist in all genuine phlegmasiæ? These cannot surely be the work of a few hours—at least we should think not—; although we know that the whole mass of the circulating fluids may undergo very striking changes in a very short space of time from the introduction of certain poisonous matters. But the two cases, it will be observed, are not strictly parallel; in the one—that of the phlegmasiæ,—the relative proportions of the solid and fluid elements of the blood is decidedly altered; whereas, in the other—that of poisoning—it seems to be rather the mode of their admixture or combination that is vitiated.

Well now, supposing that our readers agree with us in these views, they will at once perceive the grounds on which we impugn M. *Forget's* assertion that the type and therefore the treatment of a disease are not necessarily affected by the cause which may have induced it—that for example, pneumonia (or rather bronchitis) caused by the inhalation of an irritating gas is in every respect the same disease as when it occurs in the usual way. The truth of the assertion is moreover contradicted by the results of practice. It may be laid down as a rule of very general application that all suddenly-induced inflammatory diseases will not bear large depletion so well—or at least that the system suffers more therefrom—as those which are developed spontaneously. The reason is obvious;—not to allude to other circumstances, the blood is more or less altered in the one case, and it can scarcely be so in the other. Besides, in the case of an inflammation induced by the direct agency of any acrid application, we should remember that the primary action must be on the nerves of the part, and that the subsequent vascular disease must be, in a greater or less degree, the result of this. Such however is not the case with ordinary spontaneous phlegmasiæ.

We therefore repeat that M. *Forget* is, it seems to us, quite in error when he supposes that the character of a disease is not influenced by the cause which produced it. If our view be correct, we need scarcely say more as to the great practical blunder of teaching that the treatment of a disease, when it prevails as an epidemic, is necessarily the same as when it occurs in isolated and sporadic cases.

But a truce to this digression. We must now proceed to give an abstract of our author's observations on the prominent features of the epidemic meningitis, which he has so elaborately described.

*Symptoms.*—The earliest and most striking symptom, as may be supposed, was severe headache. The character of the pain varied much in different cases; being sometimes lancinating, pulsatory, boring or constricting; at other times subsiding every now and then a minute, and then recurring with increased intensity, almost like the blows of a hammer, or as if a nail were driven into the brain. In some cases the headache was the only well-marked symptom up to the time of death; but this was certainly not often the case; for usually it subsided in the progress of the disease, and delirium or coma supervened for some time before the fatal termination.

Rachialgia was the next most common symptom; and as it is not so frequent and 'banal' in a variety of complaints as headache, M. *Forget* regards it as "un signe pathognomonique de notre epidemie." The pain was sometimes along the whole spine; but in every case was much more severe over the nape of the neck than elsewhere. Often it was so acute that the patient lay in a state of almost tetanic rigidity, from fear of aggravating his sufferings by moving his head or body. Occasionally there was genuine tetanic spasm; the neck or the whole trunk being then to a certain degree drawn backwards, as in opisthotonos: in such cases the jaw also was partially locked.

In not a few instances there were convulsive contractions of the limbs recurring every now and then: twitching of the tendons also was of not unfrequent occurrence in the advanced stage of the disease. Delirium generally supervened in the second stage; and was often followed by coma before the fatal issue. It is unnecessary to particularise any of the symptoms derived from the state of the sensory organs, as there was nothing peculiar in this respect in the epidemic. One occurrence deserves notice—to wit, that in a very considerable number of M. *Forget's* cases there was a well-marked eruption of *herpes* on the lips and sometimes on the chin.

The most frequent disorder of the digestive organs was vomiting. Generally it was present at the commencement of the disease, and was apt to recur occasionally during its progress. It seemed to be owing rather to a sympathetic irritation, than to any actually morbid state of the stomach itself. The bowels were usually constipated at the same time; though this state was not unfrequently followed by diarrhœa towards the close of the disease.

The condition of the pulse varied exceedingly; very often it was as slow or even slower than in health at first, becoming afterwards more or less quickened though seldom full, and perhaps also irregular or intermitting.

We give the following case to illustrate the general characters of the disease.

A young man, when admitted into the hospital, presented the following symptoms:—great prostration of strength, sunk appearance of features, restlessness, thirst, intense headache over each temple, great pain along the spine, rapid pulse, urine deep-coloured. Six days previously, after drinking freely of beer, he had been seized with vomiting, headache, and symptoms of feverishness. The vomiting had continued up to nearly the period of his admission into the hospital; but it had ceased then. He was bled and ordered to have tartarised lemonade frequently. Next day (27th Feb.) the report states that he had been delirious during the night, but was now composed and could answer questions. He complained of pain all over him, and of great stiffness along the back and neck. His tongue was much parched; the pulse was 108 and irregular. The blood drawn yesterday was 'plastique.' He was ordered to be freely cupped along the spine, to have cold lotion applied to the head, a poultice to the epigastrium, and a solution of gum for drink. In the course of the evening of this day, comatose sub-delirium came on, and the rigidity of the spine was greater than before. He was again bled, and had a dose of calomel occasionally. On the 28th, it is stated that he had been delirious all night, and had passed a stool unconsciously: 20 leeches were ordered to be applied to the temples, the use of the calomel to be continued, and mercurial ointment to be rubbed in along the spine. Next day he was nearly in the same state: the head was shaved, 20 more leeches were applied to the temples, and the rest of the treatment was continued as before. On the 2nd of March, we find that he lay in a state alternately of coma or of delirium; the tetanic stiffness of the back continued; there were frequent twichings of the muscles of the limbs, and occasional jerkings of the tendons. Next day he died.

*Dissection.*—"Between the arachnoid membrane and the pia mater—which was highly injected—there was a layer of concrete pus covering almost the entire upper surface of the brain: it was most conspicuous along the grooves in the course of the blood-vessels. The cerebral substance appeared to be nearly normal, and there was no effusion in the ventricles. The spinal marrow seemed to be sound, except towards the cauda equina, where there was a layer of purulent matter quite similar to what was found on the surface of the brain. There were patches up and down of redness on the mucous surface of the stomach and intestines: the bladder was full of dark-coloured urine."

This case may be taken as a specimen of the general character of the epidemic. (We cannot dismiss the report, without pointing out to the English reader the dangerous error—so common among French practitioners—of continuing sanguineous depletions so long, and pushing them so far, in such diseases as that we are now considering. It is only in the first stage that they can be safely employed; afterwards we must trust to other remedies, such as cold to the head, blisters to the neck, the use of combined saline aperients and diuretics, &c. In all such diseases we should strenuously recommend the physician to promote a copious flow of urine: there is perhaps no evacuation, the state of which indicates so well the course and probable termination of the malady as this.—*Rev.*)

M. *Forget's* next case terminated favourably under the vigorous employment of free and repeated bleeding, general as well as local. But how can he, a clever man, presume to say that the "meningitis in this case had certainly passed on to suppuration." Surely the mere obstinacy of the malady and the tendency to repeated relapses are not proof sufficient, as he supposes, of such a position. So much for the extravagant length to which dogmatism will go!

In some cases, although the symptoms were by no means urgent or severe, the patient quickly and unexpectedly sank. In one of M. *Forget's* patients, a young woman—who had been only two or three days ill, and not alarmingly so, who had been walking in the ward of the hospital on the evening before—while in the act of rising to take drink, fell back and expired. The *post-mortem* appearances were by no means satisfactory to account for the fatal event. In another case, the attack had a remarkably apoplectiform character; occasionally the disease exhibited much of the appearance of *delirium tremens.*

The fatality of this epidemic seems to have been great; for out of 40 cases, treated by M. *Forget,* there were 22 deaths.

Our author, as may be supposed, details with great minuteness the necroscopic characters of the disease. But as these were quite the same as are usually met in cases of ordinary sporadic meningitis, we shall pass them over. His remarks, however—brief and imperfect as they are—on the state of the blood which was drawn deserve notice. These are his words :—

"It was usually plastic,—(what exactly is the meaning of this epithet?)—fibrinous, and without any appreciable alteration of its physical properties. In several cases we observed a *slight buffy coat,* of greater or less consistence; but never that thick and firm buff (couenne) which we find in most serous inflammations; nor yet that diffluent, loose, and mucus-like buff, and that dissolved state of the coagulum which characterise, *it is said,* septic diseases, or, to use the language of M. *Piorry,* the 'septico-hemies.' It would have been interesting to have applied those microscopic and chemical tests which have recently been 'mis en honneur' in the examination of diseases; but such criteria were not necessary 'à l'etablissement de nos convictions;' as all the other phenomena were amply sufficient to our mind to determine the decidedly inflammatory character of the epidemic."

There is a good deal in this short paragraph to comment upon, and we are tempted to make a few observations. We must premise our remarks by reminding our readers that M. *Forget* belongs to the Broussaian school, and is not very friendly to the resuscitation of the humoral views which have lately been introduced by M. *Andral,* and which we have explained so largely in the recent numbers of this Journal. Indeed he wrote a public letter not long ago to his distinguished countryman—vide No. 70 of the *Medico-Chirurgical Review* for Oct. 1841—and received an answer from him on this very subject.

Well then, it will be observed that M. *Forget* himself admits that the blood drawn from patients affected with the epidemic meningitis was, so far from being uniformly buffy, only *slightly so* in certain cases. He may call it plastic and fibrinous, although, as already said, we scarcely know the meaning of these words; but certain it is that it was not buffy, in the usual acceptation of that word. M. *Forget* seems to have been aware that many of his readers might be disposed to regard his "epidemic meningitis" rather as a species of fever attended with marked cephalic symptoms—just as the *grippe* or influenza is a pyrexia accompanied with more or less severe bronchitis—than as a genuine and essential inflammation, or phlegmasia; and he therefore somewhat artfully adds that "the *couenne* or buff was not diffluent, and mucus-like, nor was the coagulum in a loose dissolved state, as we find in septic diseases." True; but the blood is not necessarily in a dissolved state, nor even is it much changed in any respect, in many fevers. So M. *Andral* has expressly stated, and so the unprejudiced experience of every physician will testify. As we have commented at considerable length on this very subject in our notices of M. *Andral's* researches, we need not dwell much upon it at present. Suffice it therefore to state that in many of the *pyrexiæ*, the blood seems to undergo very little change, at least in its outward and physical characters; whereas in all the genuine *phlegmasiæ* there is always an increase in the relative proportion of the fibrine. Now this is a very important circumstance to attend to; as it is an axiom, we believe, of very general application that the system will never bear sanguineous depletion so well when there is not, as when there is, an excess in the proportion of this very element. It will not do for M. *Forget* to tell us that he does not require the assistance of any of those analytic means, which have been recently "mis en honneur," to enable him to determine whether a disease be "franchement inflammatoire" or not. An enlightened physician will gladly avail himself of every rational means to direct him in his diagnosis and treatment, and above all he will not neglect the opinion of such a man as his countryman *Andral*, who has so wisely kept himself apart from the dogmas of any particular *school* in medicine.

But we must be now drawing this article to a close. It would be needless to dwell long upon the treatment followed by M. *Forget*, as it is strictly Broussaian, and our readers will know that we are anything but favourable to the heroic practice of the great *reformateur*. Our author seems to have a great aversion to calomel, "cette deplorable panacée," not only in this, but seemingly in most other cerebral diseases. He adjures his brethren to tell him how often they have witnessed any good effects from the employment of this remedy, "par lui-meme," in the acute hydrocephalus of children, or in the encephalitis of adults. The answer to such a question is very simple: no sensible man, at least among English practitioners, ever dreams of trusting to any single remedy exclusively, "par lui meme," in the treatment of an active inflammation or fever. He, who does such a thing, is not a physician, but a mere experimenter. This little expression—par lui-meme—dropped so accidentally from the pen of our author, has a vast deal of meaning in it, if kept in mind when we endeavour to appreciate the merits and demerits of the French school of medicine. It lies at the bottom of a great deal of their practice, and is, as we have so often endeavoured to explain, the source and fountain of most of their practical blunders. We again repeat that there is not an acute disease in the whole nosological catalogue which ought to be treated with one single remedy, whether this be bleeding, calomel, purgatives, &c. &c. The skill of the talented physician is to use various means, all tending more or less directly to the same end although working differently, yet so artfully associated that each may assist and promote the sanative action of another. Let us take the case of any phlogistic disease, as, for example, synocha, rheumatism, or acute dropsy.

Generally speaking, no experienced physician will trust for the cure of these maladies to the use either of purgatives, or of sudorifics, or of diuretics. The chance is that he will begin by ordering a certain quantity of blood to be drawn from the arm before he resorts to any of these medicines; well knowing that by so doing not only will the severe symptoms be immediately relieved, but the action of these very remedies will be vastly promoted. How often will a gentle aperient act most efficiently after blood-letting, when the bowels had resisted patent purgatives before; and every practical man knows well the marvellous diuretic and diaphoretic effect of a dose of sweet nitre or of opium, when the system has once been relaxed from loss of blood.

Such are the considerations which induce us to object *in limine* to M. *Forget's* testimony being admitted as any authority as to the curative properties of calomel in cerebral diseases. This, like every other remedy, may be, and no doubt is abused in a multitude of cases; but he, who refuses to have recourse to it, in the treatment of acute hydrocephalus, wilfully deprives his patient, in our opinion, of one chance of recovery—notwithstanding M. *Forget's* alarms about "stomatites desesperantes," "diarrhées rebelles," and various other disastrous consequences induced by this "abominable remede!" Besides calomel, he seems to have the fear of all purgatives constantly before his eyes. "Not daring," says he, "to employ evacuants by the superior passages, I naturally had recourse, when emollients were insufficient, to the administration of laxative lavements, prepared with milk and honey, oil, senna tea, salt, &c.; and very often I had to congratulate myself, when diarrhœa came on, that I had not resorted to more active remedies."

His remarks upon the use of opium are much more judicious; and as he pays a compliment to one of the greatest ornaments of British medicine, we must give a few lines of the original passage.

........ "When meditating upon the writings of one of my favourite authors, the illustrious *Sydenham*, I had been struck with the success which, it appears, he derived from the administration of opium in various diseases attended with cerebral distress, in malignant fevers, in small-pox, &c. At the same time, I remembered the very evident advantages which I had obtained in my own practice in several cases of delirium, which had resisted the use of antiphlogistic remedies. I resolved therefore to make a trial of it in our epidemic, and the results have certainly surpassed my fondest hopes. When the inflammatory stage was once fairly subdued by bleeding and other antiphlogistic remedies, and yet such symptoms, as headache, delirium, or spasms continued, the effects of opium were in many cases quite marvellous—all the distress vanished as if by enchantment. We may observe, as a remark of general application that opium will be found most useful when there is a morbid exaltation of the nervous functions, and least so—perhaps even hurtful—when there is coma, paralysis, or other symptoms of prostration (affaissement)."......

"The results of my practice overthrow, I must admit, in some degree the classical ideas which I entertained as to the action of opium: it is so generally admitted that this medicine is not admissible in inflammatory diseases, and especially in those of the encephalon. But, after reflecting upon the subject, I have been able to explain it rationally to my own mind. This explanation is implicitly comprised in the precept laid down by *Sydenham*—viz. that, when the inflammatory element of the disease has been subdued by the use of antiphlogistic remedies, there is no danger of the symptoms being aggravated by opium, if judiciously administered. Whatever may be thought of this theory, it is sufficient for us to certify the facts which we have observed in practice. We only regret that this "inspiration" (!) did not come to our minds sooner; for we might have availed ourselves of it for practical purposes on very numerous occasions. But the relations of cause and effect have been so manifest that we—sceptical as we are on the score of therapeutic innovations—do not hesitate to publish our observations, as the expression, if not of a discovery, at least of a most useful resûscitation."

This long paragraph contains about one grain of good practical sense amidst a bushel of unmeaning pompous verbiage—announced certainly "avec trop de complaisance"; but for that single grain we are thankful, as it shews to the most prejudiced how much sterling information may be derived from the writings of the older physicians, who knew nothing of the "grandes decouvertes" of modern times. We much doubt, however, whether the "grand observateur (*Sydenham*)" who has written so much and so well on the influence of what has been called the *medical constitution* of the atmosphere, &c. would agree with M. *Forget* in regarding the late epidemic meningitis at Strasbourg as essentially and in every respect the same disease, and requiring exactly the same mode of treatment, as any sporadic and casual case of inflammation of the membranes of the brain and spinal marrow. In our opinion, the two cases are indeed "alike, but (often) oh! how different."—*Gazette Medicale.*

### Can the Future State of the Milk be predicted during Pregnancy?

M. *Donné* is of opinion that it *can;* and in a very simple manner—by examining the state of the viscid, yellowish secretion which very generally, he says, may be squeezed out of the breasts of pregnant women, and to which the name of *colostrum* has been given. This imperfectly-formed milk varies much both in quantity and in its physical properties in different cases; and M. *Donné* thinks that "he has ascertained that there is a very uniform relation between the nature of this fluid secreted during pregnancy, and the milk that is formed after delivery; or, in other words, that the examination of the colostrum and of its principal characters, enables us to predict what will be the condition of the mammary secretion, its quantity and its essential properties."

The following are the chief conclusions to which his enquiries have led him:

1. In some women, scarcely a single drop of the *colostrum* can be made to exude from the mammæ during pregnancy. When this is the case, the secretion of the milk afterwards will "presqu' à coup sur," be scanty after delivery, and probably insufficient for the infant.

2. When the *colostrum* is abundant, but thin, watery, or like a thin mucilage, and does *not exhibit any striæ of a yellow-coloured, thick and viscid matter,*—the italic letters are M. *Donné's*—the milk afterwards formed is almost always poor, and not nutritious.

3. When the colostrum is—in the eighth month of pregnancy for example—moderately abundant, so that a few drops can be obtained from the mammæ without difficulty, and especially when it is found to contain distinct striæ* of a yellow-coloured thickish matter, we may almost confidently predict that the milk will be sufficiently copious, and of good quality.—*Conseils aux Meres, &c. Paris,* 1842.

*Remarks.*—We confess that we have our doubts whether experience will warrant the importance of the signs mentioned by M. *Donné*. He is one of those zealous, enthusiastic men, full of cleverness and hope, who are apt to arrive at

* With the assistance of the microscope we often discover that the colostrum is rich in milky globules, already well formed, of a good size, without any admixture of mucous globules, and that it contains also a greater or less quantity of granular corpuscles.

positive conclusions rather more quickly than we slow-footed Englishmen quite approve of. But it is always well to know what our neighbours are thinking of and doing; as not a few useful hints may occasionally be derived from the very errors which they are apt to fall into.—*Rev.*

---

### On the Qualities of the Milk, and the Means of ascertaining them.

We need scarcely say that the old practice of judging of the qualities of a nurse's milk, by looking at a drop of it on the thumb-nail, is not to be depended upon; although some medical men still continue this practice with the view, we suppose, of satisfying popular prejudices.

Milk, we know, consists of several distinct constituent parts; some of them are dissolved like sugar in water, while others are in the solid form and float through the fluid as minute particles or globules. The *soluble* ingredients are principally 1, the caseum—the basis of cheese—2, a peculiar sort of saccharine matter; and, 3, various saline substances; the *insoluble* are only of one kind; viz. the fatty portion which produces butter. Milk may therefore be regarded as an emulsion or *looch*, in which the caseum, sugar and salts are dissolved, and the oil suspended through it in fine particles.

These various ingredients cannot be distinguished by the naked eye; but if a drop of milk be put on a watch-glass, and examined with a microscope of about 300 magnifying powers, we shall at once perceive a multitude of rounded, transparent, pearly-looking grains, floating in a limpid fluid: these are the globules of the milk, and it is they which constitute the butter obtained by churning. In pure unadulterated milk, we observe no other substance but these globules; varying much in dimensions, but all having the same general characters. It is therefore to be regarded as an unfavourable circumstance, if there be present any other particles besides the proper milky globules—such as we find under certain circumstances which we shall presently mention.

The richness, and therefore the nutritive quality, of the milk, is pretty exactly represented by the number of globules which it contains; and in proportion as these abound, so usually does the quantity of the caseum and the sugary matter also.

It is therefore obvious, that the microscopic examination of the milk affords much very useful information; it only requires a little experience in the use of the instrument to satisfy any one of this. The difference between the milks of different women is sometimes most striking; in one there is an immense number of globules, all regular, well-formed, and of a good size; while, in the other, they are small, sparse, and look like fine dust diffused through the liquid.

M. *Donné* assures us, that he has repeatedly compared the results obtained by microscopic examination of milk with those which chemical analysis of the fluid affords, and that they always agreed in their general characters: as a matter of course, there was more numerical exactitude in the latter way; but this extreme nicety is not required for the purposes of practical medicine.

The quantity of cream that forms upon milk is a simple and very useful means of determining its richness; this plan is scarcely applicable in the case of women. The cream is nothing but the coalescence of the milky globules, which, from being lighter than the medium in which they float, rise to the surface. M. *Donné* says that, according to his experiments, a healthy woman's milk yields about 3 per cent of cream, while that of the ass gives one or two only, and that of the cow from ten to fifteen, or even twenty. This experiment is easily made with the aid of graduated tubes: but, however useful it may be as an auxiliary means, it should never supersede the employment of the microscope.

*The Milk becomes thinner by remaining in the Breasts.*

A distinguished chemist, M. *Peligot*, has shewn, by a multitude of ingenious and very satisfactory experiments, that the longer that the milk sojourns, after being once secreted, in the breasts, the more thin and watery it becomes. He says, that if the product of one *milking*—that is, all the milk which a cow, for example, yields at one time—is divided into three portions, by being received into three different vessels, the first milk is thinner than the second, and the second than the third, this being the most substantial of all.

This fact, indeed, is not unknown to many nurses, who have observed that the milk that first flows from their breasts in the morning, when perhaps they have not suckled during the whole of the preceding night, is thinner and more watery than what comes afterwards.

*Formation of the Milk: the Colostrum.*

We have already said that a milky fluid begins to be secreted by the mammæ, long before the birth of the child.

It is found on examination to contain milky globules which are more or less perfectly formed, united together in small masses, or heaps, by means of a mucous matter—and also corpuscles of a peculiar nature which I have called granular bodies. The composition of this first milky secretion does not alter immediately after delivery, and the colostrum, therefore does not all at once become perfect milk. It becomes more abundant, it fills the mammæ, but we readily perceive by its yellowish colour and its oleaginous appearance that it is not genuine authentic milk.

The name of *colostrum* is therefore still retained; and it is generally believed to have slight purgative properties on the infant, and thus to promote the evacuation of the meconium.*

If such be the case, it is obvious that those children who are not suckled by their mothers, require some aperient medicine after birth more than those who are.

It is only after the milk fever has passed over, and after the infant has drawn the breast several times, that the mammary secretion acquires its characteristic properties, that it loses the oily and other strange elements, and no longer retains the viscid consistence and the yellow colour, which it had at first. Even after the change to true milk has taken place, it will be found with the microscope to exhibit for several days a small quantity of the proper granular particles of the colostrum; the proportion of those gradually diminishes until, about the sixth or eighth day, they are no longer appreciable.

In some women, however, the milk does not lose the admixture of these granular corpuscles for weeks or even months, so that its secretion is never in a state of perfect purity. This condition—which, be it remembered, cannot be detected by the naked eye of the most practised observer—it is very important to ascertain, more especially when we have to select a nurse, for suckling. The following are M. *Donné's* observations on this subject.

"The presence of granular corpuscles in milk after a certain period is unquestionably to be regarded as an irregular and morbid condition; for we observe they are produced and become more and more numerous when the health of the nurse is deranged by disease, whether this be general or of the

---

* The meconium consists in a great measure of the intestinal mucus—the elements of which may be recognised with the aid of the microscope—and also of a certain portion of biliary matter.

mamma itself. Thus, under the influence of fever, or of an engorgement of the gland itself, the milk which may previously have been quite free from them, will often be found to exhibit their presence.

The effect of the change is in general very speedily observable on the health of the infant; it exhibits all the signs of being imperfectly nourished; it is puny, peevish, and uncomfortable; the bowels are generally relaxed, and the stools are seldom healthy."

.............. "The alteration of the milk," adds our author, "by an admixture of the elements of the colostrum, is one of the conditions which will be found to be most generaly coincident with the sickly state of infant health."

*Chapped Nipples.*

M. *Donné* is of opinion that this most troublesome, and often most distressing complaint is, very often at least, connected with an altered state of the milk itself. The milk will, in many such cases, be found to be poor and watery, not very abundant, and to contain more or less of mucous matters. The child, being imperfectly fed, and finding difficulty in drawing the milk, pulls at the nipple more than usual; and perhaps at the same time its saliva is more saline than usual, and thus contributes to increase the irritation.*—*Conseils aux Meres, &c.*

---

### ADULTERATION OF MILK WITH CEREBRAL MATTER.

There has been no little stir of late among the inhabitants of Paris in consequence of a statement having made its appearance in many of the journals, that the *laitiers* of that milk-consuming metropolis were in the habit of *re-creaming* skimmed milk by mixing with it a certain portion of sheep or calves' brains made into a sort of thin paste or emulsion. The Academy, as in duty bound, had the subject elaborately discussed before them, and not a few learned memoirs have been read and written upon it. One of the best modes of detecting this clever cheat—we say *clever*, for it would seem that the adulterated milk, independently of its not being positively deleterious, is quite free from any unusual smell, taste, or appearance—is to examine the suspected fluid with the microscope viewed under a magnifying power of from 300 to 500 powers we may readily observe, blended with the ordinary globules of milk, pieces of tubes, or torn membrane or blood-vessel, very different from the amorphous greyish masses which milk is apt to exhibit after boiling.—*Gazette Medicale.*

---

### ON SUICIDE.

It is a melancholy fact that the frequency of suicide is, alas! too often in proportion to the extent of civilisation to which a people has attained. The remark indeed might seem almost paradoxical, were we not painfully aware that, when we speak of civilisation, we generally think only of intellectual and little, if at all, of moral advancement. As long as only one set of our faculties is improved

---

* The chapped state of the nipples may be, and we have no doubt is, in many instances, connected with some defect in the state of the milk itself; but this is certainly not always the case. The application of a weak solution of the sulphate of zinc, or of the tincture of catechu or kino, immediately after each act of suckling, will often speedily cause the fissures to heal; while the infant has at no time seemed to have suffered from the inconvenience to the mother.

by education, we shall often find that but little good is effected; or, at least, that the good, such as it is, is frequently counterbalanced by a proportionate amount of mischief. A celebrated character of the present day has wisely remarked in reference to the education of the lower orders, that if you do not blend religious with secular instruction, you are merely making them more *clever devils.* There is much sound wisdom in this pithy observation, as every one, who has watched the manners of the population of a large city, or paid even a very superficial attention to the history of bye-gone times, will at once admit. The mind of man is a good deal like his body in this respect. No one need be told that by far the most important rule for preserving the health of the latter is to give every part its due and regular exercise. It will not do to exercise one limb to the exclusion of the others, or stimulate any one function more than its fellows. A man's arm may be made more brawny by the long-continued use of the sledge-hammer or paviour's beetle; but the general health is not thereby improved; so far from this being the case, there is very often induced by these very occupations a tendency to heart disease, or to rupture, or to weakness of the back. Or, to take another example, the digestive energies of the stomach may be increased by the regular use of bitter infusions and tinctures, and a person be thereby enabled to consume a much greater quantity of food, and to extract too the nutriment from it, than any person who does not provoke his appetite. But then is not the consequence of such repletion very generally a plethora, or morbid accumulation of fat, or some other ill that lays the foundation for early decay?

Health consists not in the overaction of one part but in the consentaneous exercise of all parts of the body. Observe the effects of those exercises that call into play all the muscles of the limbs and trunk—for example, riding on horseback or swimming. Not a part then or sinew but is summoned into action to balance the body in the one case and to push it forward in the other. Those exercises on the other hand, which require an excessive straining of one part only, are generally injurious in the long run. Many a youth at Eton and Cambridge lays the foundation for cardiac mischief in their feats of boat-racing; and we need scarcely say that the celebrated opera-dancer is usually more early decrepid than other folks who have not pirouetted so bravely on the light fantastic toe.

Well; and so it is in the same way with the mind. If one set of its faculties be exercised unduly, while others are neglected, a vitiated and unhealthy state of things is the consequence. A few brilliant feats of cleverness may be displayed —and even these are often merely illusory and transient—but no fine and healthy harmony can be thus obtained. A man may become a walking dictionary of knowledge, and yet remain a poor unhappy discontented and perhaps withal a knavish fellow. He may study mathematics until he reason every spark of poetical feeling out of him; or he may so luxuriate in the high heavens of a poet's fancy that he utterly unfits himself for the every-day concerns of this nether world. It is not by an exclusive education of one faculty—however astonishing the results that may be thus obtained—that a truly *great* mind can be reared; nor is it by the exclusive cultivation of the intellectual powers, while that of the moral feelings is neglected—that a truly *great* and *good* character can be developed. The same law, as we have already said, holds good with respect to the mind that does with respect to the body—all its powers, and properties, and faculties must be equably and consentaneously exercised, if we hope to keep it in a healthy and continued vigorous condition. If the reasoning faculties only are cultivated, the character becomes cold, calculating and passionless; if imagination have its full swing on all occasions, unchecked and unbridled, the character will be visionary and unsteady; and if the moral faculties, even those whose natural bias is to virtue, be indulged in overmuch to the neglect of the intellect, the character, though in many respects very estimable, will be weak, easily led aside, and too often unhappy and discontented.

Dr. *Krugelstein* was in a clear-seeing, sound-thinking mood when he wrote the following remarks :—

"I am convinced that a healthy organisation and a due apportioning of the corporeal and mental energies are the only true conditions of a sound judgment —which is the result of a perfect equilibrium and constant harmony among the various faculties of the mind. According to this view, the physician will regard the predominance of any one faculty or set of faculties very differently from what is too generally done; and far from being ready to acknowledge the superiority of that mode of education which can develop what are called such brilliant results, he sees nothing in such a system but the precursory symptoms of coming disease. The further that we deviate in the education whether of mind or body from the obvious intentions of Nature, the more we shall find ourselves to be at fault."

The history of suicide, and of the light in which it has been regarded in different countries and in different times, deserves some attention.

The ancients, generally speaking, do not appear to have condemned it; and there is no positive or express denunciation of it either in the Old or in the New Testament. *Saul* fell upon the point of his sword, and destroyed himself; and yet he was buried and *David* lamented over him. *Achitophel,* on finding that the rebellion which he had instigated proved unsuccessful, hanged himself, after making his will—this proves that his voluntary death did not bar the validity of the act—and was buried in his family burial-place. *Christ* has nowhere alluded to the commission of suicide; and, in relating the death of Judas, none of the Evangelists express any opinion as to the deed. Josephus however expressly mentions suicide as a crime: "This crime," says he, "is condemned by God, and deserves punishment; it is for this reason that we have instituted the custom of casting the bodies of those who have destroyed themselves out of their houses, and leaving them exposed until sunset, although we do not refuse burial even to our enemies." Dr. *Krugelstein* says, but upon what authority we know not, that the practice, although not sanctioned by the inspired writings, was introduced by the Rabbins, just as the refusal of the ordinary rites of burial has been recognised in many Christian countries, although there is no warrant for such refusal in any part of the Bible—except perhaps in one passage of Jeremiah, where the prophet threatens King Joachim that he shall be buried as an ass— *i. e.* that he shall be cast out of the gates of Jerusalem.

The Stoic philosophers seem rather to have approved than condemned the act, at least in many cases. *Epictetus* says distinctly: "Before all things, remember that the door is always open. Be not more timid than a child, who leaves the fire whenever he gets tired of it. If you remain, do not be making complaints. If it does not suit you to bear pain or distress, Jupiter has left a door open to you; why not take your leave, and cease to accuse the Gods."

*Ælian* tells us that it was quite customary for very aged people, who thought that they could no longer serve the state, to make room for the rising generation by self-destruction: "Consuetudo est apud Ceos ut ii, qui senio plane confecti sunt, tanquam ad convivium se mutuo invitent, aut ad quoddam solemne sacrificium conveniant, et coronati cicutam bibant, cum sibi ipsi conscii sunt, se ad promovenda commoda patriæ inutiliter amplius esse, animo jam ab ætate delirare incipiente." Such a set of silly old geese!

*Montaigne* tells us that at Marseilles the public authorities always kept a poisonous compound, prepared from the hemlock, which was given gratuitously to any person who had previously satisfied the Council of the Six Hundred as to the motives which impelled them to wish for death.

Scarcely any two nations have agreed in their regulations respecting suicide. The Romans seem to have taken but little notice of it, except in the case of soldiers. *Frederick* the Great however refused to ratify the sentence of a courtmartial, which had condemned a soldier to imprisonment for having attempted to commit suicide: he said that no one in the perfect possession of his senses, ever made an attempt on his own life, and he ordered the soldier to be handed over to the doctors to bleed and physic him.

*Napoleon* took a far sounder view of the matter: he was well aware that the commission of suicide was not, as many men in the present day would have us to believe, a proof *in se* of insanity. His celebrated proclamation to the army on this subject deserves to be known by all.

*Order of the day, St. Cloud,* 22 *Floreal, an. X.*

"The grenadier Groblin has committed suicide, from a disappointment in love. He was in other respects a worthy man. This is the second event of the kind that has happened in this corps within a month. The first Consul directs that it shall be notified, in the order of the day of the guard, that a soldier ought to know how to overcome the grief and melancholy of his passions; that there is as much true courage in bearing mental affliction manfully, as in remaining unmoved under the fire of a battery. To abandon one's self to grief without properly resisting it, and to kill one's self in order to escape from it, is like abandoning the field of battle before being conquered.

NAPOLEON.
BESSIERES."

In this as all the other proclamations of this great man, a strong appeal is made to the sense of honour in his army: there is never an allusion to any higher obligations than those of being a good citizen and a good soldier. Be it remembered, however, that wilful suicide is a crime not merely of a *civil* but of a moral nature.

Formerly it was a common practice to inflict all sorts of indignities on the bodies of the poor wretches that had taken their lives. In some instances, indeed, this seems to have had some effect in deterring others. For example, we read that when *Tarquin* ordered that the bodies of those, who flung themselves off the Capitoline Hill, should be nailed on crosses and exposed to public view, the threat very manifestly checked the Roman gentry and rabble from the "perilous leap;" and a like effect was produced, we are told on the women of Marseilles, who at one period were quite *distinguées* for their suicidal propensities. Such means are all very proper to intimidate weak minds—for others would not be affected by them—when any imitative or epidemic phrenzy of self-destruction makes its appearance among a set of people; but we must seek for other correctives against the evil as it shews itself in the usual run of cases.

"Let governments," says our author, "educate and instruct the people, let them watch over their manners and habits, let them strive to oppose the debauchery, the intemperance, the gambling, and other evils of our age, let them —if possible—check the perusal of immoral and licentious books, and the exhibition of spectacles of vice upon the stage, let them relieve the distresses of poverty and disease, and not utterly neglect and cast off such persons as have become the victims of melancholy and grief."

Noble words! and no less true than noble. A little further on he breaks forth into this eloquent appeal. "How can we possibly calculate all the circumstances which may have impelled an unhappy being to take his own life? Who will flatter himself that he can know all the doublings and windings of thought, and all the secret feelings, which may have been working for a length of time in his breast? Where is the man that will be so bold as to say, 'Had I been in the place of that man, I should certainly not have acted as he has done'? There must always be much obscurity and much uncertainty hang over this melancholy theme in almost every case; and all that it is permitted us to do is to lift our hands and hearts to Heaven in prayer and say, "Oh! Lord, lead us not into temptation."

Is Dr. *Krugelstein* quite correct when he says that "all physicians are agreed in considering the act of suicide as the result of insanity"—in some, we hope many, cases it is so; but surely not in all. Of late years *Esquirol* and *Marc* have written valuable works on this subject; but, however clever these works may be, we (the Rev.) should be unwilling to receive implicitly the conclusions of French writers upon so delicate a subject; there is too pervading a spirit of materialism, and of the necessary consequences which must attend such a belief, in almost every branch of their literature, to suit our English notions on questions which are partly medical and partly ethical. *Gall* carried his fancies so far at first as to believe that there is an *organ* in the base of the brain *of self-preservation*, and that in suicides this is unusually small. Subsequently he admitted his error, and came to the conclusion that every case of premeditated suicide is an act of mental alienation—occasioned very often by a thickened state of one or more of the cranial bones, and the shrinking of the subjacent portion of the cerebral matter.

That cerebral disease exists in some cases of suicide no one will call in question, but few, we should hope, will receive the opinion of *Gall* on this subject. Suicide in almost every case is rather a mental than a bodily disease; and by far the most usual causes are either violent passion, or intense or protracted grief, or a weariness of spirits—too often the result of worn-out physical energies—or the strange but acknowledged influence of mere imitation. One common feature that will apply to 99 cases out of a 100 is, that the mind of the victim was, in some respects at least, ill-regulated and unequally poised: there must have been an excess of some faculty or emotion, and a deficiency probably at the same time of other faculties or emotions. If all classes of society, under the various trials and afflictions of life, could but feel the importance—and act too up to the feeling—of that sublime prayer, "It is the Lord's will; let Him do what seemeth good unto Him;" we should hear less of men and women opening with their own hands the "bloody house of life," and rushing into the presence of their Judge, "unhouseled, unanointed, unanealed."

Let it not be imagined that we totally reject the influence of bodily indisposition or pain as tending to induce suicidal propensities. No one, who has experienced the depressing effects of continued dyspepsia, or of dyspnœa—especially when this is at all connected with cardiac derangement—or of certain cephalic or abdominal disease, can hesitate to admit that the state of mind much depends upon the condition of the bodily health.* But then it will never do, even in such cases as these, to attribute every thing to the physical cause; else how do we see that the same diseases often exist in other persons, without being necessarily accompanied with any thoughts of self-destruction? The secret consists in this, that in the one class there is an ill-balanced, ill-directed state of mind, impatient of affliction and disappointment, discontented and repining; while in the other class there is more of cheerful submissiveness, and more of that quiet enjoyment, which springs from a "mens conscia recti." That there are however occasionally cases, in which so thick a cloud of darkness has settled around all the faculties and energies of the mind as to plunge it into the very depths of the most wretched despair, is alas! but too true—witness poor *Cowper* for example; but his case was most indubitably one of decided insanity—owing perhaps to cerebral disease, which, by-the-bye, must have been wofully aggravated by his mode of life, more especially the non-indulgence of sexual passion.

---

* With no less truth than beauty, our mighty dramatist hath said—

———— "We are not ourselves,
When Nature, being opprest, commands the mind
To suffer with the body."————

Dr. *Krugelstein,* after enumerating a host of bodily ailments, which, he thinks, may be predisposing causes of suicide, and dwelling at considerable length on the effects of youthful passion and intemperance, as too often instigating the mind to this fearful act, comments at length on the apparent influence of mere old age in some unhappy instances. He seems to think that the uneasiness of breathing, which in advanced life is so often connected with an ossified state of the rib-cartilages and incipient disease of the heart and great blood-vessels, may induce the suicidal propensity. The decay of the mental powers, too, and its not unfrequent accompaniment of querulousness and fretful apprehension—"fears are in the way"—make them weary of the life which they soon must quit. But let us hope, for poor humanity's sake, that the mind is then really in a state of imbecility or even actual insanity, when self-destruction is attempted in old age. How admirably Horace has described the infirmities of old age in the following passage:—

> Multa senem circumveniunt incommoda, vel quod
> Quærit et inventis miser abstinet ac timet uti,
> Vel quod res omnes timide, gelideque ministrat,
> Dilator, spe longus, iners, avidusque futuri,
> Difficilis, querulus, laudator temporis acti.

It is almost unnecessary to say that the mere force of imitation—however strange this may seem at first sight—has much to do with the frequency of suicide at different times. Certain it is that, if there is anything unusual either in the means resorted to or in other circumstances connected with the act of self-destruction in one case, we may almost confidently predict that we shall hear of one or more similar instances soon. The fear of posthumous indignities has been known, as already mentioned, to check this imitative mania.

It has been said that the juices of certain plants have the effect of inducing a suicidal propensity: the *spondylium heracleum* is used, we are told, for this purpose by the natives of Kamschatka.

Dr. *Krugelstein* concludes his memoir by the following remark:—

"My object has been to endeavour to convince governments, insurance companies, &c., that there is great injustice in looking upon suicide as in every instance a voluntary act, and at least that no opinion should be ever given until all the circumstances of each individual case are narrowly examined."

The mention of insurance companies here alludes to the circumstance of some of these associations declaring all policies of life insurance void where the parties have destroyed themselves.—*Encyclographie des Sciences Medicales.*

---

### Hysteria; Catalepsy; Metastasis of Hearing, &c.

A young unmarried girl, whose health had been long delicate, became subject to violent hysterical fits, in consequence, it seemed, of the irritation produced by a seton in the side—which had been inserted for the relief of obstinate pleuritic symptoms. The fits usually came an hour or two after eating. On one occasion she entirely lost her power of speech, so that she could not articulate a single word—this state lasted for three days; and then the speech returned after an hysterical seizure. Not long afterwards she experienced her first attack of distinctly-formed catalepsy; and from this period the paroxysms of this disease and of hysteria generally recurred several times in the course of 24 hours, the one alternating with the other. While she was in the cataleptic state, there seemed to be a complete insensibity of the entire surface of the body, so that it might be pinched or pricked without causing pain;—the limbs and body retained whatever position they were placed in; the movements of breathing were almost quite imperceptible; the pulse at the wrist could scarcely be felt at all, while that of the heart was exceedingly feeble and indistinct. This state sometimes lasted for one, two, or three hours at a time, the patient however not remaining all the time in this death-like repose; for every ten or fifteen minutes she would cough once or twice, and then a movement of some part of the body followed, or she would even suddenly start up, leap out of bed, clime upon a chair or table, &c. and there perhaps assume an attitude that was "ordinairement fatigante," but yet "toujours gracieuse:" her features on such occasions always wore an air of "douceur et satisfaction." In this state she would remain until a fit of coughing came on, or till she was removed to bed by her attendants. One curious circumstance in this, as in other cases, of somnambulism is that, although the eyes be quite closed, the person avoids with the greatest nicety any impediments in their way, and perform feats which they would not even attempt to do in their waking state. On one occasion she rushed to a window, opened it, and was on the point of throwing herself out, when she fell into a cataleptic paroxysm, with one foot out of the window, and the other resting on the ground: in this position she remained, until she was replaced in bed.

The cessation of each cataleptic fit was always indicated by the return of the respiratory movements, which gradually became more and more decided until the patient opened her eyes and recovered her intelligence.

During one of these attacks, Dr. *Duvard* observed that, when he applied his hand to the epigastrium, the countenance of the girl gave expression of decided suffering, and to his surprise he found that if he applied his mouth to this region, —or, as subsequently was the case, to the soles of the feet or to the palms of the hands—and spoke to her, she answered questions with perfect correctness, although she appeared to be utterly deaf to any auditory impressions on the ear itself. It was not even necessary that the mouth should be applied directly to the *impressionable* parts; the intervention of a stick or of a rod of iron, &c. the one end of which was applied to the epigastrium or to the palms or soles, and the other end to the lips of the speaker, did not at all prevent the patient from hearing what was addressed to her, as her rational answers to any questions put in this manner abundantly testified. Dr. D. confesses that he had often read of similar occurrences as this in books, but that he never felt inclined to attach any credit to the reports of them, till he met with the present case. . . . .

"There was no sensibility in the integuments of any part of the body, except of the epigastrium and of the palms of the hands and the soles of the feet. The skin might be pricked or pinched, the hair might be pulled out by the roots, the nostrils might be irritated, &c., without the patient exhibiting any symptom of pain or uneasiness. But if the epigastrium, or the palms or the soles were even tickled gently with a feather, she immediately drew back, and her features indicated that something offended her. When the ball of a charged Leyden jar was applied to any of the sensitive parts, she at once made a violent start, and sometimes awoke up in a moment; but, strange to say, she remained quite motionless and passive even when the jar was discharged upon any other part of the body. (!)

The senses of taste too and smell were singularly perverted as well as that of hearing. The most pleasant or the most nauseous substances might be placed upon the tongue, and the nostrils might be stuffed with assafœtida or tobacco, or irritated with hartshorn or æther, without apparently any sensation being excited; but if any sapid or strongly odorous substance was placed upon or approached to any of the sensitive regions, the countenance of the patient at once indicated that she was aware of it, and, if questioned, she answered at once whether it was pleasant or not, and could even sometimes tell the name of the substance used. When snuff was put upon her hand, she at once began to sneeze, although it produced no effect when pushed up the nostrils." (!)

There was, however, no transport of vision nor yet any *clair-voyance* with Mademoiselle *Melanie*.

Dr. D. subsequently lost sight of his patient; but he heard that the hysterical and cataleptic attacks had become much less frequent and severe than formerly. All medical treatment seemed to have been quite unavailing.—*Gazette Medicale.*

*Remarks.*—Cases like the preceding are certainly very *droll*, to say the least of them; and it will certainly puzzle the most knowing savant of the present day to account for some of the phenomena which they exhibit. There is one way, indeed, of solving the difficulty, and that is to deny *in toto* all the alleged facts, and to call all such statements by the very comprehensive appellations of *humbug*, and tomfoolery.

But be it remembered, that excessive incredulity may be quite as ridiculous as extravagant credulity. It is no good reason why we should utterly disbelieve any alleged fact, because we cannot explain its occurrence, or because certain circumstances or phenomena connected with it seem at variance with our ordinary experience. There are so many mysteries in the phenomena of life, which are, and will probably always be, utterly inexplicable, that it is no easy thing to say where our belief is to stop. Far be it from us to lend a complacent ear to the extravagancies which have been so absurdly exhibited of late years on the subject of animal magnetism: they are too ridiculous to last but as a "nine month's wonder;" but this is no sufficient reason for flatly denying, or smartly ridiculing, the reports of all such cases as the preceding one.

---

### Deafness Cured by the Endermic use of Morphia.

As there is no set of complaints, which medical men are so puzzled to know how to relieve as those connected with hearing, we are always ready to receive a hint that may prove useful in some cases, provided it comes from a respectable man who is not an advertising aurist. The name alone of Dr. *Hoebeke*, the narrator of the following case, is a sufficient guarantee for its truthfulness.

A lady had become so deaf after an attack of fever that she could not distinguish a word, unless it was bawled into her ear by applying the mouth close to it. But along with the deafness there was always an incessant noise in the ears —at one time like the hissing of boiling water, at other times like the roaring of a hundred voices together—which was often so distressing as to cause headache and confusion of ideas:—these feelings were always worse when the head was on the pillow. There was a quantity of wax in the ears; but no relief was obtained when it was removed. Nothing irregular could be perceived either in the ears themselves or in the throat. Leeches were applied behind the ears, and emetics and purgatives given; but no relief followed. Supposing that the symptoms might be dependent upon some anomalous state of the nervous apparatus, a blister was applied behind each ear, and the excoriated surface was sprinkled with half a grain of sulphate of morphia. By the next day the noise and deafness on the left side had quite ceased, and on the right were much abated:—the headache, too, had disappeared.

As the unpleasant feelings still continued on the right side, a second blister was applied and treated in the same manner as before with morphia:—the success was decided, and the patient was quite freed of all his annoyances.—*Archives de Med. Belge.*

*Remarks.*—The medical man often meets with cases very similar to the preceding, and, in not a few, every thing they try is unhappily without avail. How far the remedy proposed by M. *Hoebeke* will answer in many of these, we must leave to the experience of our readers to decide.

We may state that we have occasionally in such cases used with advantage a belladonna plaster applied either to the temple, or behind the ear of the affected side. Many of the complaints of hearing seem to be exceedingly influenced not only by the state of the general health, and especially by that of the stomach, but also by the quietude or irritability of the temper, as well as by the varying conditions of the atmosphere. No wonder then that we are so often foiled in relieving them.

---

### THE SURGICAL PRACTICE AT THE HOTEL DIEU.

In one of the late numbers of the French Medical Gazette there is a statistical report for 1841 of the surgical practice of Professor *Roux* at the Hôtel Dieu in Paris—the largest hospital, it is well known, in that metropolis.

The reporters preface their statements rather oddly; they say: "With the exception of a certain number of persons, whose complaints were not sufficiently grave to warrant their remaining in the hospital, almost all have exhibited some peculiarities worthy of notice. To avoid, however, extending this report too far, we have limited it to the great surgical questions, to unusual operations, to those which M. *Roux* has *instituées*, or which he has in some degree made his own by the perseverance and skill with which he performs them—all of which circumstances tend to give to his practice a peculiar degree of interest, and to attract a vast multitude of patients to his wards."

The total number of patients admitted under his care, during the 12 months, was 1441—of whom 1303 were males and 138 were females.

The operations performed were as follow:—

| | Cases. | Deaths. |
|---|---|---|
| Amputation of the thigh | 8 | 5* |
| Amputation of the leg | 5 | 3 |
| Amputation supra-malleolar | 1 | 0 |
| Amputation of the fore-arm | 2 | 1 |
| Amputation of the metacarpal bones | 4 | 2 |
| Amputation of the fingers | 7 | 0 |
| Resection of the elbow | 3 | 1 |
| Resection of a rib | 1 | 0 |
| Extraction of sequestræ | 2 | 0 |
| Trepan | 2 | 2 |
| Cancer of the lower lip | 3 | 0 |
| Epulis | 1 | 0 |
| Cancer of the cheek | 1 | 0 |
| Section of the genio-glossi for stammering | 1 | 1 |
| Cancer of the tongue | 2 | 1 |
| Cancer of the velum palati | 1 | 0 |
| Staphyloraphy | 3 | 0 |
| Excision of the tonsils | 4 | 0 |
| Excision of an osseous cyst on the upper jaw | 1 | 0 |
| Hernia, crural | 6 } | 5 |
| Hernia, inguinal | 3 } | |
| Fissure of the anus | 18 | 0 |
| Fistula, recto-vaginal | 1 | 0 |
| Anus, imperforation of, *Littre's* operation | 1 | 1 |

---

* In one case both thighs were amputated.

| | Cases. | Deaths. |
|---|---|---|
| Anus, at umbilicus | 1 | 1 |
| Amputation of the penis | 1 | 0 |
| Amputation of the testicle | 3 | 0 |
| Lithotomy | 4 | 3 |
| Lithotrity | 2 | 1 |
| Excision of the mamma | 6 | 0 |
| Strabismus | 6 | 0 |
| Cataract | 41 | 0 |
| Artificial pupil | 1 | 0 |
| Varicocele | 5 | 2 |

With several others of minor importance.

The total number of deaths that occurred after operations was 34. If we add to this 56, the number of cases which proved fatal spontaneously, either from the severity of the accident sustained, or the gravity of the existing disease, we find the entire mortality to be 91 during the twelvemonths.

By far the most frequent cause of death after the operations was purulent absorption and the consequent contamination of the whole system, the induction of hectic fever, &c.

If we may judge from the result of one year's experience, it would seem that cold weather is decidedly unfavourable to the success of great operations: nearly two-thirds of the deaths occurred during the months of January and February.

Erysipelas, it deserves to be noticed, was by no means very prevalent during this year: the absence of this most pernicious visitant may perhaps account for the comparative smallness of the mortality during the warm months.

There is very little interest in the separate details of this report; it is meagre and unsatisfactory. As we have heard so much for the last year or two of the *brilliant results* of myotomy, applied to various diseases, we select the following notice of M. *Roux's* practice in a case or two of stammering.

*Stammering; Section of the Tongue; Failure.*

The Reporters state with not a little justice that, even before the results of surgical interference in the treatment of this complaint were at all ascertained, a variety of operations had been proposed and adopted by different men. Unfortunately for them all, they seem to have now fallen into complete oblivion.

M. *Roux*, as a matter of course, was called upon to test the real merits of the cutting method of treating impediments of speech. In one case he divided merely the frænum of the tongue—in an old man, 60 years of age—which was perfectly free in its movements. This man certainly stammered most shockingly, all the muscles of his face being often thrown into convulsive spasms, when he made an effort to speak. No sooner was the frænum divided, than "avec beaucoup de netteté il se confond en remercimens;" and he naturally expressed his astonishment that he should have been allowed to remain all his lifetime without relief. The cure seemed to every one at the time to be "brilliant and complete;" when, behold, he began to stammer as bad as ever, and in this state he remained during all the time that he was in the hospital.

In another case, M. *Roux* divided the *genioglossi* muscles at their insertion into the lower maxilla: here too the stammering ceased at the time; but speedily returned; and, when he left the hospital, he was no better than before. He subsequently put himself under the care of M. *Colombat*, and derived considerable benefit—like the youth we alluded to in our last Number, who, after having had his mouth and tonsils most unnecessarily cut by Mr. *Yearsley*, has been quite cured by Mr. *Hunt*.

---

### M. RAYER ON PHTHISIS.

The principal facts embodied in a very elaborate memoir on this subject, which this distinguished physician recently read at the Academy, are these :

1. Tuberculous phthisis is, of all chronic diseases, the most widely spread among men and animals.

2. In man, and in other mammals, the tuberculous matter may be readily distinguished from recent pus, which is always loaded with granular globules. In birds, the characters of tuberculous matter are less distinct. Foreign substances introduced artificially into the lungs, or into the muscular flesh (of birds?) occasion the formation, not of a white, opaque and globular fluid, like pus, but of a dry, yellow, unglobular matter, whose physical properties resemble a good deal those of genuine tubercles.

3. Purulent matter in mammiferous animals,—more especially in the horse,—undergoes, after a long sojourn in any organ, successive transformations, so as sometimes to assume the appearance of tuberculous matter.

4. Pulmonary tubercles in man, and also in quadrumanous animals, have usually a greyish colour.

5. The central softening of tubercles cannot be attributed to inflammation. They never exhibit any globules of pus. But the softening of the circumference or exterior of tubercles is certainly favoured by the inflammation of the adjacent tissues; and there they are almost always blended with pus.

6. The yellow matter, which is found in hydatidic cysts—after these have burst—in ruminating animals, has some analogy with the tuberculous matter of the *pommeliere* of the cow; but the cysts, that are filled with this matter, almost always contain the debris of the hydatidic pouch, and sometimes also a certain quantity of pus.

7. The cretaceous concretions—which are principally composed of carbonate and phosphate of lime—occasionally observed in the lungs of man as well as of the lower animals, should not be considered, as hitherto they have been, as being the last modification of tuberculous matter: they are often in the human subject, and very often in the horse, the residue of a small purulent deposit.

8. In several animals there are formed in the lungs certain *verminous* and *glanderous* granulations, which must be distinguished from those of a genuinely tuberculous nature.

9. In quadrumanous animals, and also in birds which have been brought from hot climates to this country, the development of phthisis is found to be at its maximum point of frequency, and almost to the exclusion of other chronic diseases. The same morbid formation is nearly as common in animals brought from an arctic to a temperate climate. We must not forget to take into account the change of diet at the same time, as having no inconsiderable influence in both sets of cases.

10. Phthisis is of rare occurrence among domesticated solipedous animals, and is still rarer among carnivorous quadrupeds and birds. Yet, in spite of the preservative effects of a strong constitution and of an animal diet at the same time, tuberculous disease is found frequently to affect the lions, tigers, &c. that are kept in our menageries.

11. Among the carnivora, the dog, and among the solipeda, the horse, is less subject to tubercles than to cancer—a disease which used to be regarded as peculiar to man.

12. Among ruminating animals, and especially in the cow tribe, the presence of phthisis is often associated with the existence of hydatids; but there seems to be no relation either as to transformation or succession between hydatids and tubercles.

13. The fatty degeneration of the liver indicates usually the presence of phthisis in man, and of general obesity in birds.

14. The alteration of the bones, which we observe in monkeys affected with tuberculous disease, appears analogous to the swelling and spongy softening of the bones in scrofulous phthisical children. Similar changes have been noticed in the bones of some of the carnivora, which have been brought from hot climates to this country.

15. If the frequency of pneumonia and the rarity of phthisis in the domestic dog seem to contra-indicate any relation between these two diseases, it is not so in the cases of milch-asses and cows: for in these animals the deposition of tuberculous matter is almost invariably co-existent with a progressive chronic pneumonia.

16. Phthisis is unquestionably hereditary; but it is almost never congenital, even in its earliest or rudimentary state.

17. In phthisical persons, the semen, contained in the vesiculæ seminales, is found to contain few or no spermatic animalculæ.

18. The ulcerations of the larynx, trachea, and bronchi have not the signification in man as they have in the lower animals. In the former, their existence is almost always a sign of pulmonary phthisis—occasionally of syphilis; in the quadrumanous tribe of a general tuberculous affection; and in solipedous animals almost invariably of glanders.

19. When pneumo-thorax exists in a case of phthisis, spots of mouldiness have been known to form on the altered pleura, as they have been sometimes observed in the air-sacs of birds affected with tubercles. In all such cases the formation of these vegetable matters is a *secondary* phenomenon.

M. *Rayer*, at the close of his memoir, alludes in a particular manner to the influence of captivity and domestication on the production of tuberculous disease among the lower animals. This cause may be aptly compared to the influence of imperfect and unwholesome nourishment and of impure residence, the prolific causes of phthisis in man.

---

### Development of Cryptogamæ in the Bodies of Animals: Treatment of Tinea.

MM. *Rousseau* and *Serrurier*, in a memoir on the diseases of the organs of the voice, which they communicated to the Academy in 1839, stated that, in a parrot which had died of laryngeal and pulmonary phthisis—a common disease in tropical birds—they found, between the intestines and the vertebral column, a sort of false membrane, on which there was a greenish pulverulent mouldiness, so light and so feebly adherent, that it might be blown away with the breath. They have observed a similar phenomenon in the bodies of some of the mammalia and once also in a tortoise.

M. *Deslongchamps* has more recently confirmed the complete accuracy of the statement.

M. *Dumas* mentioned that Dr. *Gruby*, after long examination of the subject, has come to the conclusion that one of the varieties of *tinea*, the t. favosa, is owing to the development of a vegetable formation under the epidermis, similar to that produced by the fermentation of yeast, and to what has been occasionally found in saccharine urine.

M. *Schoenlen*, of Berlin, had anticipated Dr. *Gruby*'s observations, having published a paper on the same subject in Müller's Archives, some time before.

(It will be observed that M. *Rayer* alludes to the occasional occurrence of mouldiness in the neighbourhood of diseased tissues in the preceding article.)

It would certainly seem that there is something peculiar in the ætiology of such a disease as tinea. It is often so entirely local, and seemingly so completely unconnected with constitutional derangement—although most cutaneous diseases unquestionably are—that we should not be at all surprised to learn that it is caused by the development of certain entozoa, whether these be of an animal or of a vegetable nature.

To pass, however, to the treatment of tinea, we must caution our readers never to allow themselves to be misled by the reports of rapid cures alleged to have been effected by this or by that remedy. We firmly believe that no plan of treatment can "juguler" (to use M. *Bouillaud's* phrase,) the disease; for it seems to have a certain course to pass through, or, at all events, the affected parts seem to require a certain time before they recover their healthy state. After having the hair closely cut, and the parts well cleaned and softened with poultices, &c. the application of a solution of the sulphate of zinc, or of copper—the strength of the solution varying according to the effects produced—is one of the best remedies we know. An oil-skin cap should always be worn, as the parts are thus readily kept moistened, without a frequent renewal of the lotion. On the whole, we prefer fluid to unctuous applications; they are more cleanly, and we can more easily watch their effects.

Some ointments, however, are in many cases decidedly useful: that made with the ioduret of sulphur is a good one; a mixture of equal parts of sulphur and of golden ointment is also very useful occasionally.

The great objection to the nitrate of silver, is that of discolouring the integuments, so that we cannot judge of its action, until the cuticle comes away. A weak solution of the corrosive sublimate, the black-wash, Goulard lotion, &c. are all occasionally useful. It is generally well to soften the scalp well during the night with any simple unctuous application, such as pure hog's-lard.

The state of the secretions from the bowels and kidneys should be always punctually attended to. We are very partial to the daily use of small doses of the sulphate of magnesia, and of the nitrate or of the sulphate of potash, in the infusion of roses, to which the vinum colchici and tinctura lyttæ may be added. All constitutional irritation is thus removed, and a derivation, so to speak, is caused to the bowels and kidneys by the use of the remedy, if continued for two or three weeks at a time.

The state of the cutaneous functions, too, should not be neglected: the daily use of the tepid affusion over the head and body can never do harm, and will often tend to accelerate the cure of this as well as of many other diseases of children.

---

### OXALIC ACID A REMEDY IN MUCOUS INFLAMMATIONS.

At the scientific meeting recently held at Turin, M. *Nardo* communicated the results of various experiments made during the last 12 years with this salt. According to his observations, it possesses antiphlogistic properties superior to those of all other vegetable acids, and has at the same time a peculiar sedative action in allaying the severe pains which generally attend inflammation of the mucous membranes, as in the various forms of angina, gastritis, aphthæ, &c. The dose of the acid should be about one decigramme (a grain and a half?) dissolved in any simple vehicle. M. *Nardo* has seen the most satisfactory results in the aphthous affections of children from the judicious administration of oxalic acid, either alone or combined with peppermint or some vegetable astringent: he considers it to be a contra-stimulant sedative.—*Journal de Chemie.*

---

### Tincture of Creosote.

| | | |
|---|---|---|
| Take of | Creosote .. .. .. .. .. .. .. | 24 grains. |
| " | Alcohol .. .. .. .. .. .. .. | 4 drachms |
| " | Tincture of Cochineal .. .. .. | 2 drachms. |
| " | Peppermint Oil .. .. .. .. .. .. | 12 drops. |

This tincture forms an admirable remedy against the pain of toothache in many instances, if a piece of cotton moistened with it be applied upon the decayed tooth. A few drops of it mixed with water makes an excellent lotion for preserving the gums in a healthy condition.

---

### M. Orfila on the Absorption of Chemical Substances into the Blood, &c.

During the last two or three years, we have repeatedly called the attention of our readers to the very interesting researches of the distinguished toxicologist of Paris relative *to* the absorption of certain metallic salts, more especially those of arsenic, antimony and copper, into the animal system, and the means of detecting their existence in various organs of the body after death.

We learn from the following letter, addressed by him during last April to the Academy of Medicine, that he has been prosecuting his enquiries on this hitherto imperfectly examined point of pathological and physiological science; and we earnestly recommend all practical men not to neglect a diligent acquaintance with such subjects, as it is far from being improbable that the therapeutic action of many active medicines may become much better understood, and thereby the practice of physic be improved, by the discoveries of modern chemistry.

"The following results I have obtained," says M. *Orfila*, "from a great number of experiments, the details of which will shortly be published.

"1. The diluted sulphuric, nitric, muriatic, and oxalic acids, are absorbed and may be detected in the urine.

"2. The same acids, even in their concentrated condition, are also absorbed; but this probably occurs after their becoming blended with the watery juices of the stomach and bowels, the secretion of which is increased by the contact of such stimulating agents.

"3. The absorption of the salts of lead, bismuth, tin, zinc, gold and silver cannot be disputed, seeing that we find in the liver and also in the urine of animals, which have been poisoned with those substances, the metals which form their bases. All these metals are discoverable by the same analytic process; viz. carbonising the viscus with strong nitric acid, and treating the residue with aqua regia or with nitric acid.

"4. The salts of mercury also are absorbed and carried by the blood into all the viscera of the body. To prove this, we have only to treat one of the viscera of an animal, which has been poisoned with a mercurial salt, with aqua regia, and to pass through the solution thus obtained a current of chlorine gas; or what is still better, to carbonise the viscera with a strong acid in a closed vessel, and to treat the residue with boiling aqua regia.

"5. I have also detected in the viscera—more especially in the substance of the liver and in the urine—traces of the sulphuret of iodine, of nitrate of potash, of alum, of ammonia, and of sal ammoniac.

"The results now stated complete the suite of researches which I proposed to undertake on the subject of the absorption of poisonous matters derived from the mineral kingdom; they serve to establish beyond all doubt the correctness of the views which I explained in my former communications relative to the absorption of the salts of arsenic, antimony, and copper."—*Gazette Medicale.*

*Remark.*—This subject of the direct absorption of metallic salts and other chemical agents into the system, and the penetration of all the organs of the body with them, deserves especial notice from the practical physician as well as from the medical jurist.

---

### HINTS ON THE MANAGEMENT OF INFANTS.

There is so much good sense in a great part of M. *Donné's* work that we have been tempted to make several extracts from it, or rather to put upon paper our own ideas on the same subjects, and blend them with those of our author. There may be no novelty in them; but they are not for this reason the less useful.

Let us, for the present, take one point connected with the *feeding of infants.* Whatever be the age of the infant, it is always of great utility that the food be given at regular periods, as nearly as possible equi-distant from each other, so that the stomach of the young creature is neither left too long empty, nor oppressed and unnecessarily loaded. A sufficient interval of time must therefore be allowed to elapse after each repast, to enable it to be digested and then discharged from the stomach before another be given. To put fresh food into the stomach, while it is still more or less filled with what had been taken before, is inevitably to disturb the process of assimilation, by which the food is converted into chyle for the nourishment and growth of the body.

Now this very simple circumstance—so simple that the experience of daily life dictates it, and withal so important that the health cannot be maintained without due attention to it—is, strange to say, very seldom attended to in the rearing of infants. How generally do we observe that, instead of giving food to them only at regular and stated periods, no sooner do they begin to cry, than they are immediately applied to the breast, although perhaps not half an hour had elapsed since they had had it to their little heart's content. It surely needs not the authority of a medical man to assure any one that such a practice cannot be wholesome or right.

We might derive a useful lesson on this point by watching the conduct of the lower animals towards their young. We do not, for example, observe that the cow will allow the calf, or the sheep the lamb to be tugging at the teat, whenever the young creature seems to wish it. It is only now and then, and at certain intervals, that they are allowed to satisfy themselves with the "milky food." We may be assured that nearly the same law applies to all animals, and that what holds good in the case of the calf and of the lamb holds good of the human infant also.

In one respect only have the dumb creatures in our fields the advantage over us, and it is this: with them, *instinct*—that truly marvellous gift of an all-wise and beneficent Creator—is an unerring guide, while man's boasted *reason* is, alas! often either asleep, or is leading him astray.

But it may asked, how often then should the infant have the breast? The following few directions will, we think, suffice to direct our readers on this very important question. The younger that the infant is, the more frequent is the desire, and necessity for food. During the first weeks of life, the breast may be given every two hours or so in the day—less frequently at night. As to the quantity which the child should have at each time, the nurse will, as a general remark, find that it may be allowed to continue sucking until it is satisfied. When once this is the case, all that is given afterwards, is only oppressive to the stomach; and it is well known that the little creature usually gets rid of the superfluity by bringing it up. When the child is very weak, it may be necessary to give the milk more frequently and in smaller quantities at a time; but in ordinary cases an interval of about two or three hours should be allowed to elapse between each meal.

The child should, however, seldom be awaked for the purpose of giving food. It is an old saying, that " a sound sleep is almost as nourishing as a full feast;" and the remark is especially true of infants. They require a great deal of sleep, and they are always refreshed and strengthened by it. As long as a child sleeps, we may rest pretty well assured that Nature does not need any fresh supply of food; and therefore to disturb it merely because the regular period, at which otherwise it would have been applied to the breast, has arrived, is not only unnecessary but hurtful. The exceptions to this remark occur very rarely indeed, and then only in the case of infants who are exceedingly weakly, and who should therefore be regularly seen by a medical man, as no general rule can be laid down that will be applicable to all.

It may be worth mentioning that, in not a few instances, the poverty of the nurse's milk seems to have something to do with excessive drowsiness of the infant. Nature appears to endeavour to compensate for the deficiency of nourishment by an extra allowance of repose. In all such cases, therefore, the medical man should satisfy himself most accurately on this point, more especially when the child is suckled by a hired nurse—who, it may be supposed, always tries to make us believe that her milk is both abundant and good. There is a simple rule which will very seldom misguide us: it is this: if the child does not thrive properly and yet sleeps a great deal, we may feel pretty confident that the food administered is not sufficiently nutritious, and therefore that some change in the diet is necessary.

The frequency of suckling should be diminished after the sixth or seventh week, or even earlier in the case of robust hearty children. Once every three hours is then quite sufficient, provided the milk be duly nourishing and the infant be allowed to satisfy its craving at each time. At the end of the third month, the breast need not be given oftener than every four hours; and so on successively, an extra hour being added to each interval after every additional month or six weeks of life. By following this system, not only is the health of the infant best consulted, but the convenience and comfort of the nurse will be greatly promoted.

How many a mother, from an overflowing affection, devotes almost every hour of every day to watching over her beloved child! She is unwilling to leave her home for even a few hours, from fear of it suffering for want of the breast while she is away. Now this is just one of those errors, which it is the leading object of M. *Donné*'s work to endeavour to get rid of, both for the mother's and the child's sake. If the latter be accustomed from its birth to have food only at stated regular times, it is altogether better in every respect that in the intervals the nurse be away, and the child be left in the care of another person. We have already explained how much the condition of the milk is affected by the state of the general health, and how necessary it is for the maintenance of this, that the nurse should take regular out-door exercise, and that her mind be kept in an equably cheerful state. Now, how can these advantages be obtained, if she be continually from morning to night with her child? Another great evil of the nurse being too much with the infant, is that the latter, by the mere smell of the milk being constantly presented to it, is often induced to desire food when it certainly does not require it, and when, therefore, it would be much better without it.

This, it has always appeared to us, is one of the greatest disadvantages attending the nurse being too much with the infant—at least until it has been accustomed not to seek for the breast, except at stated intervals. And Nature is so flexible at this early period of life that this most desirable end may easily be gained by adopting a judicious plan from the commencement.

---

### PHYSIOLOGICAL LAWS OR MAXIMS.

A second edition of Baron *Michel's* Translation of Professor *Mojon's* "Physiological Laws,"—a work which, on its first appearance in Italy, received the high commendation of *Scarpa, Mascagni, Tommasini* and *Borda*—has been published a few months ago in Paris. That our readers may judge for themselves of its contents, we have selected the following "Laws" on the important functions of Digestion, Circulation, and Respiration. They are correct as far as they go, but are very far from exhausting the subjects of which they are intended to give a condensed summary.

A useful work might certainly be got up by making Professor *Mojon's* one the basis on which to build a series of more extended remarks.

### *On Digestion.*

1. The existence of a central organ for digestion is one of the most essential characters of animal life.

(This is certainly a distinctive feature of animals—the existence of a central sac for the admission and digestion of the food. The very lowest of the Zoophyte tribe have it; and indeed the whole body of these creatures is little else but a stomach provided with a single opening. No plant, as far as we know, is similarly organised: their food seems to be invariably absorbed by the minute vessels of their extremities, and is never received into one single cell or sac.)

2. Every animal requires, for its sustenance, to introduce into its digestive organ, at certain intervals, some substance capable of affording nourishment to it.

3. For any substance to be fitted for the nourishment of animals, it is necessary that it should be of an organised structure itself, that it be soluble in the juices of the stomach, and capable of furnishing the elements of chyle.

(We here observe another important mark of distinction between animal and vegetable life; the one requires matter which has been already organised for its food, while the other derives its nourishment exclusively (perhaps) from inorganic matter.

One great end therefore served by plants, in the wonderful economy of the creation, seems to be to transmute and prepare the materials of the earth as food for animals. We know of no mineral or unorganised substance that any animal can live upon. We read, indeed, of savages eating clay and earth; but then this is merely to satisfy the cravings of hunger, when they cannot procure regular food, or it is the effect of a diseased appetite, such, indeed, as we not unfrequently meet with in some cases of chlorosis. On the other hand, matter must always (probably) be in an inorganic condition to serve as food for vegetables. It may be imagined that, in some instances, as in that of the Dionœa Muscicapa or Venus' fly-trap, plants seem to derive their nourishment from animal substances; but this is very doubtful. It is more than probable, that these very substances must first be decomposed into their elementary constituents before they can be used as materials for nourishment to the plant. This then seems to be the series of changes undergone by matter: the earth, the air and the water are absorbed by plants, and serve as food to them; by the wonderful chemistry of vital action, they are subsequently transmuted into the various products of vegetable life: these products supply the food of the greater number of animals, and these again, become the prey of other animals, which are carnivorous. All the excretions of animals are already inorganic matters; and even their bodies, when left to slow decay, are gradually resolved into a few elements, which belong to the mineral kingdom.)

4. Food cannot nourish or repair the waste of the body, until after it has undergone the action of the digestive process, and its materials become assimilated to those of the body itself.

(We cannot wonder much at this, when we consider that no mineral substance agrees in its composition with any vegetable substance, nor yet any vegetable substance in every respect with one that is animal. Some vegetable and animal substances, indeed, are very nearly alike in their composition; they consist of the same elements, but then these elements are combined in different proportions. Even in the mineral kingdom we find that a somewhat analogous law exists. All the atoms of a crystal, for example, are essentially and entirely alike; and never do we find the particles of a different arrangement—although their elements be the same—combined together in a regularly formed salt.)

5. Vegetable substances require a much more laborious digestion on the part of the stomach and bowels, to fit them for nourishment than animal substances.

(This is what we might expect; vegetable substances approach much nearer to the constitution of mineral inorganic matter than animal substances do, and we may therefore, *à priori*, suppose that they must undergo a greater change before they can be assimilated with animal bodies. Hence we find that the digestive canal in the herbivora is of much greater length than it is in the carnivora.)

6. Substances which are entirely destitute of azote,—such as oil, gum, sugar, &c.—cannot by themselves serve as food for animals, at least for those which are carnivorous.

(Every one has heard of the experiments of Majendie on this subject: the animals, when fed with these substances alone, quickly pined, and died of atrophy.)

7. Before any animal substance can be digested, it must have lost its vitality.

(The same may be said of vegetable substances. Indeed the very nature of digestion implies a complete separation of the component parts of any substance that is used for food. Milk is perhaps the article that undergoes least change; and it is well known how nearly this fluid approaches in its characters and composition to chyle.)

8. Animals, that are fed with farinaceous and other vegetable substances, are generally much fatter than those which live upon flesh.

(The chemical composition of all fatty matter approaches much nearer to that of vegetable substances than other parts of animal bodies. It contains a very large proportion of carbon and very little azote; whereas muscular fibre contains much less of the former, and more of the latter, element.)

9. All hoofed and horned animals are herbivorous.

10. The sensations of hunger and thirst are felt whenever the stomach is empty. They are more imperious in youth than in age,—because the body requires more food in early years for its growth—and in all animals that have much exercise than in those which are inactive.

11. External cold and every other influence which quickens the digestive functions, render hunger more intense. In the same manner, whatever induces a great loss of the fluids, increases the desire for drink.

(The ingenious views of the celebrated German chemist, *Liebig*—fully detailed in the present number of this Journal—on the influence of cold air, &c. on the powers of digestion in men and animals, should be attentively studied. He points out with great skill the intimate connexion that exists between the respiratory and the digestive functions, and the beautiful adaptation of the food in different climates to the desires and wants of the resident animals. This is a most interesting chapter in the harmony of animal life, which had never before been so satisfactorily investigated.)

12. Heat, rest, strong mental emotions, the sight, or even the mere remembrance, of any disgusting object, constipation of the bowels, &c. diminish the keenness of hunger. On the other hand, prolonged hunger speedily enfeebles not only the muscular strength, but also the heat of the body, and the vigor of the intellectual powers.

(On the much vexed topic of the cause of animal heat, we must again refer to M. *Liebig's* work. This distinguished writer advocates the chemical doctrine as to the cause of this vital function, and in the course of his disquisition he shews with great force the influence of the food that is used, and of the atmospheric conditions in which the animal is placed, on the development of the heat of the body.)

13. An animal, in a state of nature, is always led by its appetite to select the food that is best fitted for its sustenance. There exists a constant relation between the kind of the aliments on which the animal lives, and the peculiar dispositions of its gastric system.

14. The form of the teeth has a considerable influence on the digestion of the substances which the animal can subject to mastication.

15. The eruption of the teeth in the child takes place between the first and the fourth years. Usually they are pushed out in pairs, to the number of four-and-twenty in all. From the sixth to the seventh year, the sixteen milk or caducous teeth are successively replaced by others, so that the entire number of permanent teeth amounts to 30 or 32. The milk teeth generally drop out in the order of their eruption from the gums.

16. The configuration and arrangement of the teeth vary much in different animals, according to the sort of food they live upon.

17. In carnivorous animals, the teeth are strong, curved, and pointed, for the purpose of tearing and dividing the flesh on which they feed. In the frugivorous tribe they are sharp, flattened, and so arranged as to cut and grind the substances placed between them. In the herbivorous and granivorous tribes, they have a cuboid form, and are adapted to bruise the food by their broad uneven surfaces. In man, who is omnivorous, we observe these various forms combined together.

18. The chyme, as it is formed in the stomach, passes through the pylorus into the duodenum, where it is mixed with the bile, pancreatic and intestinal juices, and is gradually separated into chyle and feculent matter.

19. The strength of the walls of the stomach is in general inversely as the development of the masticatory organs, and the facility with which the food is digested. The solvent power of the gastric juice is also inversely as the amount or degree of the other forces which assist in the digestion of the food. The gastric juice in carnivorous animals is very different from that in the herbivorous.

20. In animals, which hybernate during Winter, the process of digestion is altogether suspended during the whole period of their lethargy.

21. The bile in the duodenum combines with the pancreatic liquor and mixes with the chymous pap. These three substances become mutually decomposed. The most soluble and nutritive portion of the chyme unites with a portion of the biliary and pancreatic juices to form the chyle, while another portion of the bile combines with the excrementitious part of the food, which traverses the intestinal canal, loses its chylous juice, and is at length voided as excrement.

22. The irritability and sensibility of the intestinal tube diminish gradually from the stomach downwards to the rectum.

23. The form and nature of the excrementitious matters are nearly alike in different animals of the same species, although they be fed on very different substances; while in animals of different species, but fed with the same food, the fæces have always a distinctive character in each tribe.

*The Organs of Circulation.*

24. The size of the heart, compared with that of other parts of the body, is greater in the fœtus than in the born child; it is also greater in short than in tall persons: ('a little body, but a mighty heart.')

25. The capacity and force of the heart are, in proportion to the rest of the vascular system, greater at the beginning than at any other period of life.

26. The heart is proportionally larger and stronger in bold courageous animals than in those which are feeble and timid: it has greater energy in the carnivorous than in the herbivorous tribes.

(A long chapter might be written on this subject. How the very language of all nations bears witness to the truth of the observation that the force of the mind and will has something to do with the state of the circulation: a strong heart, a bold heart, a faint heart, and such like, are daily expressions everywhere. No man can possibly be thoroughly courageous in danger, unless his circulation remains tranquil and little moved all the while. True, it may be said that the corporeal condition is the result of the mental. This is certainly true; but let it be observed at the same time that, with a little tact and management, a person may succeed in maintaining extraordinary courage in circumstances of the most appalling danger, if he will but attend to the state of his breathing, so as never to allow this to flag or to go on feebly and irregularly. If every now and then he will take in a deep breath, or make a strong effort to laugh or sneeze, the heart will be thereby relieved, and the circulation will go on more evenly.)

27. In mammiferous animals the volume and force of the heart are always proportionate to the development of the lungs.

28. The capacity of the ventricles of the heart in warm-blooded animals is always greater than that of the auricles. The reverse is the case in cold-blooded animals.

29. In proportion as the arteries recede from the heart, their diameter diminishes, and their number increases. The entire capacity of the arterial system increases as it is distant from the heart; for the sum of the diameters of all the arterial ramifications much exceeds the diameter of the common trunk.

30. The arterial, as well as every other, tissue becomes more indurated as the animal advances in life.

31. The contractility of the arterial parietes is proportionally greater in small than in large animals.

32. The arteries contain more blood than the veins in the early years of life, and less in middle and advanced age. There is an arterial plethora, as long as the superiority of the forces over the resistances continues; but, when the rigidity of the solids begins to be established, there is a plethora of the veins gradually established.

33. The capacity of the entire venous system is greater than that of the arterial.

34. The capacity of the veins is in an inverse ratio with the celerity of the fluid that permeates them.

35. The quantity of the blood, which is distributed to all the parts of the body, varies at different periods of life. It is, relatively to the volume of the body, more considerable in infancy than in adult age.

36. The vessels of the head receive, in proportion to the rest of the body, more blood in the early than in the late years of life.

37. When the body has attained its full degree of development, the quantity of blood in the veins increases constantly in a direct ratio with the diminution of that in the arteries.

38. During embryonic life, there is no apparent difference between the arterial and the venous blood.

39. In the human fœtus the blood does not contain any phosphatic salts; after birth, however, they exist in it: it is also found to contain then more hematosine, to acquire a brighter red colour, and be more coagulable than it was before. At the age of puberty the blood is warmer, and has a spermatic odour, somewhat like that of the sweat of the animal. In the adult, it has more consistence, and is richer in fibrine than in the youth; and in old age, it becomes paler, and has a great tendency to form different sorts of coagula or concretions. The proportion of the cruor to that of the serum is generally inversely as the age of the person.

40. The characters of the blood differ much in different classes of animals. It is more or less red in all animals which have a bony skeleton; yellow or whitish in insects and in most of the mollusca; and watery and transparent in zoophytes. In birds it is usually redder, warmer, less serous, and more coagulable than in mammals. In reptiles and in fish it is very serous, but does not readily coagulate, and is much disposed to become oily: its temperature too is but little above that of the air or water in which the animal lives.

41. In all warm-blooded animals, the circulation is found to be the more rapid, the sooner after birth that it is examined; and the rapidity gradually becomes less and less as life advances.

42. The arterial pulsations in the mother and in the fœtus are independent the one of the other; they are certainly not isochronous, although there is such an intimate relation between the existences of the two beings.

43. During the arterial circulation, the blood loses successively more and more of its albuminous and fibrinous nutritive ingredients, as well as of its heat, and becomes charged with hydrogen and with carbon: then it becomes venous.

*Of the Respiration.*

44. Respiration takes place in all animals without exception; it is effected however in very different ways, and with different degrees of activity in different tribes. The contact of the air is indispensable to every animal; for even those, which live constantly in the water, find in it a certain quantity of air, sufficient for their existence.

(Of late years it has been discovered that plants will live for a great length of time without the renewal of fresh air. It has been found that, if the pots in which they grow be covered with a bell-glass hermetically secured round them, so as quite to exclude the atmosphere, living plants may be brought over from India to this country without risk, so that, when transferred to our hot-houses, they will thrive perfectly. How long animal life will continue under such circumstances is not easily ascertained. We read of frogs, &c. having been found imprisoned in trunks of trees, &c., where they must have remained a length of time, without losing their vitality.)

45. All animals, which have a heart or central organ of circulation, respire by means of a particular apparatus; and this apparatus has always an intimate and reciprocal relation with the organs of circulation.

46. In all animals, in which the respiration is localised, the special organs for the performance of this function have a fine vascular structure, which is spongy and capable of presenting to the air an extensive surface. In the mollusca, crustacea, and the red-blooded worms, the respiration is effected by means of circumscribed bronchia. In fish, the central apparatus of respiration consists of two aquiferous bronchia, and in insects by tracheæ over every part of the body. In the true zoophytes, the medusæ, and the polypi, the whole of the body seems to act as a respiratory organ; no special apparatus is discoverable.

47. In general there is an exact relation between the activity of the respiration and the energy of the other functions, as the circulation, the digestion, &c.

48. Amphibious reptiles and serpents have the power, by reason of their peculiar organization, of suspending at will their respiration, without stopping the circulation of the blood.

49. The lungs of warm-blooded animals are passive in the act of inspiration, but they aid the movement of expiration by a power which is proper to them.

50. Whatever quickens the circulation, increases the rapidity of the respiratory movements. There is in general a very marked relation between the quantity of oxygen absorbed during respiration in a given space of time, and the vivacity of the movements of the animal.

51. In the adult human being, the respiratory movements vary from 18 to 24 in the minute—or in the ratio of about one movement to every five beats of the pulse.

52. In mammals and in birds, the nature of the respiratory movements has much to do with the expression of their voices—their cries, their singing, their yawning, &c.

53. The quantity of oxygen absorbed by the blood during respiration is greater during Winter than during Summer.

(This must necessarily be the case when the air is cold, and therefore dense—provided the number of the respirations continue about the same. Now the greater amount of the oxygen absorbed requires a greater quantity of carbon, derived from the food, for its combustion or combination within the body. Hence there must be a greater amount of caloric developed. It is by following out this chain of reasoning in a variety of ways that M. *Liebig* has succeeded in throwing not a little light on some of the most disputed points of physiological science.)

54. Animals after eating absorb more oxygen during respiration than when they are fasting. The consumption of oxygen is in proportion to the need which the constituents of the blood have for combining with it.

55. In birds, the inspired air is received into a multitude of vesicles, distributed over different parts of the body. This arrangement not only renders the weight of their bodies much less, but also forms a reservoir for the purposes of breathing in very elevated regions of the atmosphere.

(It has been clearly made out by recent aeronautic experiments that the breathing is by no means so much embarrassed at very great altitudes, as was formerly supposed—provided no bodily exercise be taken at the time. In the various ascents of Mr. *Green* of late years, we have not heard either of him or of any of his companions suffering much from inspiring the rarified air at great heights in the atmosphere. A good deal therefore of the distress, so often experienced by travellers who have ascended very high mountains, must be attributed to the effect of bodily fatigue.)

56. Birds consume, in a given space of time, much more oxygen, in proportion to the size of their bodies, than quadrupeds do: this is in part owing to the rapidity of their respiratory movements.

57. Among the many purposes of respiration, one of the most important is to invigorate and renew the force of the muscles, by restoring to their fibres their exhausted irritability. It is found that the motive energy, as well as the general vital activity, in different animals, are exactly proportionate to the rapidity and force of their breathing.

(This remark is quite true of animals of one class, but certainly not of different classes of animals, compared with each other. For example, the strength and activity of most fishes are surprising, especially when compared with many warm-blooded animals; and yet their respiration is, so to speak, much more imperfect than in the latter. We might allude also to the wonderful muscular strength of many insects, especially of the coleopterous kind.)

58. The less that the respiration is fitted to restore the irritability of the animal fibre, the greater is the tenacity of the fibre in retaining this property.

(The truth of this remark is strikingly exemplified in the case of most reptiles, whose flesh it is well known, continues to exhibit contractility for a long time after death; while that of warm-blooded animals scarcely survives life.)

59. Those warm-blooded animals, which hybernate during certain seasons, are nearly in the same condition as animals having cold blood, in relation to the quantity of oxygen consumed during respiration.

---

### ACCOUNT OF THE DEATH OF THE DUKE OF ORLEANS.

This most melancholy event has excited so much sympathy and interest among all classes, not only in France itself, but in this and in other countries, that a brief account of the circumstances connected with it will be read, we doubt not, with interest by every one. The accident was a very frightful one, and the report of it is interesting in a mere surgical point of view, as shewing what amount of injury may be caused by a fall from a vehicle when going along at full speed.

On the morning of the 13th of July, about a quarter after eleven o'clock, the Duke set out for Neuilly to join the King and Queen there, before starting to take the command of the camp at St. Omer.

He was in a low four-wheeled carriage—called a *Daumont*, and which is like a large open cabriolet—a groom being seated behind. It was drawn by two spirited horses. On leaving the *Barriere de l'Etoile*, the horses, especially the leader—(were they harnessed tandem-fashion?)—became rather unruly, and the driver gradually lost command over them. Wishing to give himself more space, he turned them to the right into the *Avenue de la Revolte*, which is in a line with the St. Denis road. The Prince, observing him to deviate from the usual route, called out to his postillion; but he, although he heard the Prince speaking, could not distinguish what he said. It was probably at this time that the Prince stood up to see how things were going on. He leaped or was thrown out, and immediately fell, striking his head with great violence on the curbstone. Two gendarmes, who happened to be on the road at the time, and the owner of an adjoining small grocery shop, ran to the spot, and lifted him up. They at once recognised who it was, and immediately carried him into the shop.

While they were carrying him, he vomited the food he had taken at breakfast: this, it is well known, is a common symptom attending injuries of the head. The royal patient was stretched out upon two mattresses on a low bed, in a back room: this was about half-past eleven o'clock. On the first news of the accident, a number of medical men hurried to the spot. M. *Pasquier*, first surgeon to the King, and his son, the surgeon of the Prince, soon arrived: MM. *Blandin* and *Blache* followed almost immediately afterwards. Alas! it was but too evident from the first moment that the case was a hopeless one. We may readily conceive the grief of M. *Pasquier* (the son) who had for many years enjoyed the confidence of the Prince, had accompanied him and never left his side in Africa, and who, before being appointed his surgeon, had been his instructor in medicine. For the King, who has always wished that the education of his sons should be very complete, had made the Prince—then Duke of Chartres—study anatomy, physiology, and a little of surgery.* For six months he dissected, under the instruction of M. *Pasquier*, at the Hôtel des Invalides. Perhaps this was one reason why he was always so partial afterwards to military surgeons. He had seen them at their humane labours in the field of battle in Africa, where,

---

* *Louis Philippe* himself studied anatomy, and the simpler operations of surgery, as bleeding, the application of bandages, &c. under *Dessault*.

as everywhere else, they always proved themselves to be, as he beautifully said, "men of science and brave soldiers."

The loss of the Prince to the medical officers of the army will long be deeply felt.

*Revenons.*—The Prince was stretched out on his back, his head resting on his chest, in a state of complete insensibility and muscular powerlessness. A young German did the pious duty of supporting the head of the Prince, during the five hours that he survived. The breathing was deep, slow, and laborious; the pupils were dilated and unaffected by light; and the mouth and ears contained some blood. The pulse was small, thread-like and compressible. On examining the head, no depression or irregularity could be felt; but it was too apparent that irreparable mischief had been done to the encephalon, and that the case was one of cerebral commotion of the third and last degree, in which scarcely a single hope of recovery can be entertained. Cooling lotions were applied to the forehead, and vapour of ammonia and other stimulants to the nostrils; but all in vain; not an appearance of consciousness was shewn, but every now and then involuntary movements of different parts occurred.*

It was now about noon; and at this time the *King*, accompanied by the *Queen*, *Madame Adelaide*, the *Princess Clementine*, and followed by *Marshal Gerard*, and Generals *Athalin*, *Gourgaud*, *Rumigny*, and M. *Delessert*. The Queen threw herself on her knees at the side of the truckle-bed, where lay her unfortunate son. Never did a mother's grief and desolation break forth in more heart-rending expressions. In the midst of this scene of despair, the King alone mastered his anguish.

The state of the patient became worse and worse. Sixty leeches were applied near the base of the cranium. It was about this time that he uttered a few unconnected words in German.† He tried, too, to tear the leeches off, as if they caused him pain. Sinapisms were applied.

The breathing became more noisy, irregular, and oppressed, and the twitching movements of various parts became stronger. The lower extremities, which hitherto had been quite motionless and flaccid, became affected with a general tremor, followed by irregular, convulsive contractions. Gradually these movements became less frequent, and at length ceased, leaving the parts in a state of almost tetanic rigidity. The breathing became more and more stertorous, and the pulse became more feeble than ever.

During the whole of this time, the Queen was kneeling at the side of the bed, supplicating the Almighty to grant her dying son one moment of consciousness, and offering her own life as the price of such a mercy. Around her stood the members of her family, in a state of the most distracting despair. The grief of the young Dukes, *Montpensier* and *d'Aumale*, was most affecting; the latter constantly exclaiming, " Oh! when *Joinville* hears of this!" The King looked on this scene of affliction with a resignation which was even more touching than the louder sorrow of the rest.

M. *Pasquier* applied the cupping-glasses—*scarifiées et seches*—on the trunk,

---

* These movements had taken place some time previously, after a bleeding that had been performed before the arrival of M. *Pasquier*. The propriety of such a practice in cases of violent commotion of the brain is more than questioned by the best surgical authorities: it must necessarily increase the already existing depression of the vital energies.

† The words could not be made distinctly out. It may be that the image of his wife visited his thoughts in this hour of darkness, or that he was calling to one of his German valets, who were in the habit of waiting upon him, and whom he was in the habit of addressing in their own language.

and limbs; and hot sand was applied to the soles of the feet, and sinapisms to the ankles. The pulse rose somewhat for a few minutes; but this slight change soon ceased. At two o'clock, the curate of Neuilly, whom the Queen had called for repeatedly, arrived to administer extreme unction to the dying Prince.

The convulsive movements of the limbs became gradually more violent; the muscles appeared to be affected with a continued spasmodic agitation. The breathing became more and more difficult, the pulse at the wrist ceased, and that of the carotids could, about three o'clock, scarcely be felt. All the usual symptoms of approaching death—the pale visage, the purple lips, the half-closed fixed eyes, the rattling breath—succeeded. More than once it was thought that the prince was dead; at length one deep sighing inspiration was heard, and all was over. This was at half-past four o'clock.

The clergy were introduced, and all present fell upon their knees. Never was there a more impressive scene. In a poor back room of a small grocer's shop were to be seen the King and Queen, the Princes and the Princesses, of France, with the ministers of state and of religion, all kneeling around the humble bed on which lay the corpse of him who was the heir to the throne, while the clergyman repeated in the prayers for the dead. It was difficult indeed to say which was the most affecting of the two; the sobbings and tears of the broken-hearted mother, or the silent scarce-restrained grief of the father, in this most trying moment of deepest affliction.

*Autopsy.*—This was performed by M. *Pasquier,* the son, forty hours after death, in the presence of General Baron *Athalin,* first aide-de-camp of the King, and the following medical men, MM. *Fouquier,* first physician to the King, *Pasquier, Moreau, Blache, Blandin,* and *Destouches.* There were marks of a contusion on the right cheek and right side of the forehead, also on the back of the left hand, on the front of both knees, and on the left hip. There was a broad sanguineous swelling over the back part of the head.

*Cranium.*—The integuments being divided from before backwards along the median line, the soft parts over the occiput and thence to the forehead and temples were found to be infiltrated with blood: the infiltration was considerable in the posterior portion of the occipito-frontalis muscle. The two flaps of integuments being folded down on each side, the saw was applied in the usual place, and the cranial bones divided. The violence of the blow, with which the head came against the ground, must have been extreme, as we may judge from the severity of the injuries inflicted. The lambdoidal, the left squamous and mastoid, the sphenoid, and the two spheno-petrous sutures, were all partially disunited. The cranium was fractured in numerous places. One fracture, beginning at the *right* side of the lambdoidal suture, passed a little above the posterior and inferior angle of the parietal bone, and extended into the temporal fossa, as far as the great *ala* of the sphenoid. Another, starting from the *left* side of the same suture, divided the parietal bone, from behind forwards, along one-half of its length, and had also separated the squamous portion of the temporal from the rest of this bone. The squamous suture being disunited, this portion of the temporal bone was quite isolated, and adhered only to the soft parts. A third fracture divided in a transverse direction the sphenoid bone on the level of the sella turcica.

On removing the calvarium, the anterior and inferior portion of the cerebrum, as far back as the fissures of Silvius, was found reduced to a reddish pulp or *detritus.* There was much sanguineous effusion under the arachnoid membrane: a few drops also of bloody serosity were found in the ventricles. One of the optic nerves was fairly divided across. All the other organs of the body were perfectly sound, with the exception of the lungs, which were so much gorged with dark blood that their tissue almost resembled that of the spleen.

